# Characterization of the pathoimmunology of necrotizing enterocolitis reveals novel therapeutic opportunities

**DOI:** 10.1038/s41467-020-19400-w

**Published:** 2020-11-13

**Authors:** Steven X. Cho, Ina Rudloff, Jason C. Lao, Merrin A. Pang, Rimma Goldberg, Christine B. Bui, Catriona A. McLean, Magdalena Stock, Tilman E. Klassert, Hortense Slevogt, Niamh E. Mangan, Wei Cheng, Doris Fischer, Stefan Gfroerer, Manjeet K. Sandhu, Devi Ngo, Alexander Bujotzek, Laurent Lariviere, Felix Schumacher, Georg Tiefenthaler, Friederike Beker, Clare Collins, C. Omar F. Kamlin, Kai König, Atul Malhotra, Kenneth Tan, Christiane Theda, Alex Veldman, Andrew M. Ellisdon, James C. Whisstock, Philip J. Berger, Claudia A. Nold-Petry, Marcel F. Nold

**Affiliations:** 1 Department of Paediatrics, Monash University, Melbourne, VIC Australia; 2 Ritchie Centre, Hudson Institute of Medical Research, Melbourne, VIC Australia; 3 Department of Immunology, Hokkaido University Graduate School of Medicine, Sapporo, Japan; 4 Department of Medicine, Monash University, Melbourne, VIC Australia; 5 Department of Gastroenterology, Monash Health, Melbourne, VIC Australia; 6 Department of Anatomical Pathology, Alfred Hospital, Melbourne, VIC Australia; 7 Central Clinical School, Monash University, Melbourne, VIC Australia; 8 ZIK Septomics, Jena University Hospital, Jena, Germany; 9 Department of Molecular and Translational Science, Monash University, Melbourne, VIC Australia; 10 Centre for Innate Immunity and Infectious Diseases, Hudson Institute of Medical Research, Melbourne, VIC Australia; 11 Department of Surgery, Beijing United Family Hospital, Beijing, China; 12 Capital Institute of Pediatrics, Beijing, China; 13 Department of Pediatrics, Goethe University Hospital, Frankfurt, Germany; 14 Department of Pediatrics, St. Vincenz Hospital, Limburg, Germany; 15 Department of Pediatric Surgery, Goethe University Hospital, Frankfurt, Germany; 16 Helios Clinic Berlin-Buch, Berlin, Germany; 17 Roche Pharma Research and Early Development, Roche Innovation Center Munich, Penzberg, Germany; 18 Mater Research Institute, University of Queensland, Brisbane, QLD Australia; 19 Neonatal Services, Mercy Hospital for Women, Melbourne, VIC Australia; 20 Joan Kirner Women’s & Children’s, Sunshine Hospital, Melbourne, VIC Australia; 21 Department of Newborn Research, Royal Women’s Hospital, Melbourne, VIC Australia; 22 University of Melbourne, Melbourne, VIC Australia; 23 Murdoch Children’s Research Institute, Melbourne, VIC Australia; 24 Medicum Wesemlin, Department of Paediatrics, Lucerne, Switzerland; 25 Monash Newborn, Monash Children’s Hospital, Melbourne, VIC Australia; 26 Department of Pediatrics, Liebig University Hospital, Giessen, Germany; 27 Biomedicine Discovery Institute and Department of Biochemistry and Molecular Biology, Monash University, Melbourne, VIC Australia; 28 Australian Research Council Centre of Excellence in Advanced Molecular Imaging, Monash University, Melbourne, VIC Australia

**Keywords:** Innate lymphoid cells, T-helper 17 cells, Infant necrotizing enterocolitis, Immunopathogenesis

## Abstract

Necrotizing enterocolitis (NEC) is a severe, currently untreatable intestinal disease that predominantly affects preterm infants and is driven by poorly characterized inflammatory pathways. Here, human and murine NEC intestines exhibit an unexpected predominance of type 3/T_H_17 polarization. In murine NEC, pro-inflammatory type 3 NKp46^−^RORγt^+^Tbet^+^ innate lymphoid cells (ILC3) are 5-fold increased, whereas ILC1 and protective NKp46^+^RORγt^+^ ILC3 are obliterated. Both species exhibit dysregulation of intestinal TLR repertoires, with TLR4 and TLR8 increased, but TLR5-7 and TLR9-12 reduced. Transgenic IL-37 effectively protects mice from intestinal injury and mortality, whilst exogenous IL-37 is only modestly efficacious. Mechanistically, IL-37 favorably modulates immune homeostasis, TLR repertoires and microbial diversity. Moreover, IL-37 and its receptor IL-1R8 are reduced in human NEC epithelia, and IL-37 is lower in blood monocytes from infants with NEC and/or lower birthweight. Our results on NEC pathomechanisms thus implicate type 3 cytokines, TLRs and IL-37 as potential targets for novel NEC therapies.

## Abstract (web summary)

Necrotizing Enterocolitis (NEC) is an untreatable intestinal disease in infants. Here the authors show that human and experimental mouse NEC is associated with altered toll-like receptor expression in the intestine, enhanced Th17/type 3 polarization in adaptive immune and innate lymphoid cells, dysregulated microbiota, and reduced interleukin-37 signaling.

## Introduction

Necrotizing enterocolitis (NEC) is a severe gastrointestinal disease that primarily affects infants born prematurely and with a very or extremely low birth weight (VLBW < 1500 g; ELBW < 1000 g). Up to 11% of VLBW preterm infants are affected by NEC annually in the US^1^. NEC currently is the most common cause of death between postnatal days 15 and 60 in infants born before 28 weeks of gestation^2^. The disease entails substantial morbidity and mortality, ranging from 20–30% in confirmed cases^3^ to 65% when surgery is required^4^.

Current treatment options for NEC are limited to bowel rest, antibiotics, and supportive therapy such as blood pressure management^5^. Gut perforation occurs in ~20–50% of NEC infants, necessitating surgery^1,4^. Thus, the prognosis for NEC infants is grim and those who survive commonly have to deal with long-term neurodevelopmental and growth complications^6,7^, rendering new therapeutic strategies for NEC an urgent unmet need.

NEC pathogenesis is multifactorial, with four main risk factors: prematurity^8^, formula feeding^9^, abnormal microbial colonization^10^, and hypoxic/ischemic states^11^. However, the relative contribution of each of these risk factors is unclear, and despite decades of research, the pathogenesis of NEC remains largely elusive. Nevertheless, existing literature points to intestinal dysbiosis in NEC that results from an imbalance between pro-inflammatory mediators such as Toll-like receptor (TLR)4, interleukin (IL)-1β, IL-6, tumor necrosis factor (TNF) and IL-18 on the one hand, and protective anti-inflammatory mediators such as TLR9, IL-1 receptor antagonist (IL-1Ra), IL-10 and transforming growth factor (TGF)β_2_ on the other^12^. This imbalance leads to a vicious cycle in which excessive pro-inflammatory signaling and intestinal injury reinforce one another and perpetuate disease activity^12^.

Although many of the immune players with a role in the vicious cycle of NEC are known^12^, the sequence of events and the importance of individual mediators and cells in NEC pathogenesis remain poorly understood. Little is known about the adaptive immune system in NEC and even less about specific immune cell subsets in the preterm gut. Innate lymphoid cells (ILC), which closely resemble the T helper (T_H_) lymphocyte subsets T_H_1, T_H_2, and T_H_17 in terms of cytokine production^13^, have become increasingly implicated in inflammatory bowel diseases^14–16^. However, only very few studies have linked ILC dysregulation and neonatal intestinal pathology^17,18^. Furthermore, there is a striking paucity of omics-type approaches in NEC research.

The excessively inflammatory intestinal environment that characterizes NEC has prompted the evaluation of broad-spectrum anti-inflammatory mediators such as IL-10^19^ and TGF-β^20^ as therapeutics. IL-37 is a powerful anti-inflammatory cytokine that is inducible by a multitude of pro-inflammatory stimuli such as IL-1β, IL-18, TNF, IFNγ, or activation of TLR1/2, 4, and 9^21^. Moreover, IL-37 has a wide range of actions, including suppression of various pro-inflammatory cytokines such as IL-1, IL-6, and TNF^21^, and attenuation of adaptive immunity^22^. As a mouse homolog for IL-37 has yet to be discovered, in vivo studies use mice transgenic for human IL-37 (IL-37tg)^21^. We have previously shown that lipopolysaccharide (LPS)-induced inflammatory responses are markedly attenuated^21^ in IL-37tg mice, and that IL-37 is protective in murine colitis^23^. Taking into account additional data collected by us and others on IL-37^24^, this member of the IL-1 cytokine family has emerged as a promising therapeutic option for the treatment of inflammatory diseases of the gut and other organs.

**Fig. 1:**
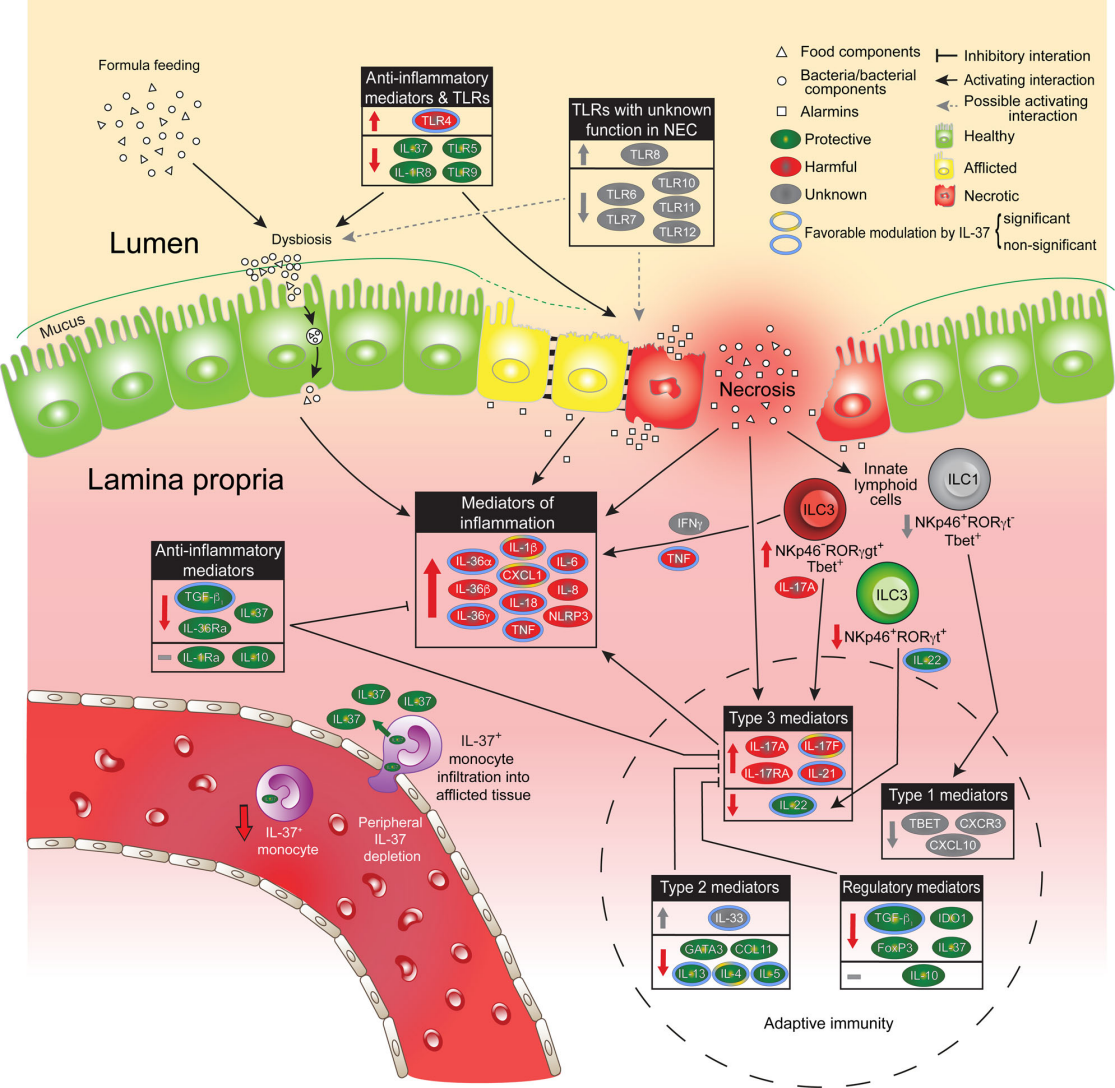
Overview of innate and adaptive immunity in NEC and of the protective effects of IL-37. Colored arrows within boxes and next to ILC designations and IL-37^+^ monocyte within blood vessel: Color indicates consequences of NEC-induced changes as protective (green), harmful (red), or unknown (gray); direction denotes increase (upwards), decrease (downwards), and no change (flat line). The green arrow on monocyte crossing endothelial barrier signifies the release of IL-37 into the subepithelial tissue. Colored rings around cytokines indicate prevention of increase or decrease, or restoration to baseline by IL-37 (i.e., favorable modulation by IL-37); no border, not affected by IL-37 or not assessed in this regard. The dashed circular line encloses changes and effects pertaining to adaptive immunity. Interactions depicted by black and gray connectors make no claim to be comprehensive; interactions relevant to the data reported in this paper are prioritized. Categorization into protective, harmful, and unknown is based on current knowledge of prototypical function; see ref. ^12^ and references in the text.

Here we explore several pathomechanistic aspects of NEC in a mouse model that uses clinically relevant triggers of the disease. As summarized in Fig. 1, we show that NEC profoundly disrupts the intestinal homeostasis of innate immunity (e.g., changes in IL-6, TLRs, CXCL-1, TGF-β, IL-36 agonist:antagonist ratios), adaptive immunity, and ILC (e.g., dominance of type 3 over type 1, type 2 and regulatory polarization) as well as microbial diversity. These mouse data are supported by studies of resection specimens and peripheral blood from human preterm infants with NEC and appropriate controls. Besides providing cross-species validation for most of the murine findings, the human data reveal an NEC-associated epithelial deficiency in IL-37 and its receptor IL-1R8, and a lower IL-37 abundance in monocytes, particularly at 2 weeks of age when NEC most commonly occurs. Accordingly, bolstering IL-37 protects mice from NEC-induced intestinal injury and mortality, and restores several (e.g., innate cytokines, IL-4, IL-17F, microbial diversity), but not all (e.g., not ILC imbalance) disruptions caused by NEC. Together, our data advance knowledge on the pathoimmunology of NEC and highlight translational opportunities that could be exploited to bring relief to young patients suffering from this devastating disease.

## Results

### IL-37 ameliorates tissue injury and symptoms in murine NEC

In order to assess whether IL-37 protects against NEC, we employed an established mouse strain that transgenically expresses human IL-37b (IL-37tg)^21^. Newborn IL-37tg and C57BL/6J WT mice were separated from dams after birth and subjected to formula feeding, asphyxia, and cold stress to induce NEC. Approximately half of each set of littermates remained with their dams and served as dam-fed controls.

**Fig. 2:**
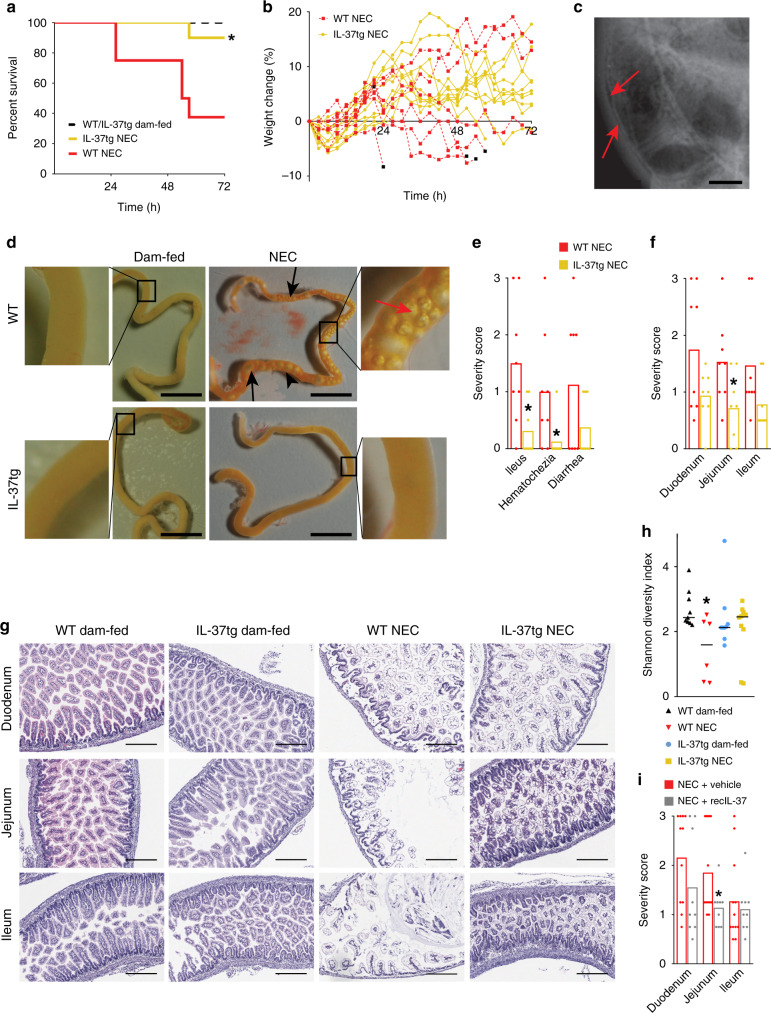
IL-37tg mice are protected from NEC. **a**–**h** Newborn IL-37tg and WT mouse pups were randomized into dam-fed or NEC groups. Dam-fed pups remained with the dam; NEC pups received 3-hourly formula feeds and cold stress and asphyxia twice daily. Data are from three independent experiments with *n* = 8 mice for WT NEC and 10 for IL-37tg NEC. **a** Percent survival and **b** percent weight change of IL-37tg vs WT mice subjected to NEC; log-rank test *P-*value: *P < 0.05. Black squares denote death endpoints. The starting weights of WT and IL-37tg pups were similar. **c** X-ray of a human infant with NEC. Red arrows indicate pneumatosis intestinalis; also note dilated bowel loops indicative of ileus. **d** Representative photographs of small intestines from mice of each experimental group immediately following excision at the time of death or experimental endpoint. Black arrows denote luminal air bubbles, red arrows pneumatosis intestinalis (gas trapped in the intestinal wall), scale bars are 1 cm, side panels are ×5 magnifications. **e**–**g** *n* = 8 pups for both WT NEC and IL-37tg NEC. Student’s *t*-test or Mann–Whitney *U* test *P*-values: *P < 0.05 for IL-37tg vs WT. **e**, **f**, **i**, **h** Graphs show measurements in individual pups as dots, bars indicate means (**e**, **f**, **i**) or medians (**h**). **e** Clinical scoring of NEC-associated parameters (0–3 scale, no to severe pathology, see “Methods” section). **f** Histological scoring of intestinal regions in NEC mice (0–3 scale; see “Methods” section, Supplementary Fig. 1 and panel **g**). **g** Representative photomicrographs of intestinal sections from each of the four groups, e.g., showing a healthy mucosa in WT dam-fed mice (score 0), mild vacuolation in an otherwise relatively healthy mucosa in IL-37 NEC jejunum (score 1), an advanced disease in WT NEC duodenum (score 2) and severe NEC with tissue disintegration in WT NEC ileum (score 3). Magnification ×100; scale bars indicate a 300 µm. **h** Shannon diversity index representing the intra-group microbiome diversity (alpha-diversity) within each of the four experimental groups. *n* = 9 pups for WT and IL-37tg dam-fed, 6 for WT NEC and 10 for IL-37tg NEC; non-parametric *t*-test (Monte Carlo permutation) *P*-value: *P < 0.05 for WT dam-fed vs WT NEC. **i** Newborn WT pups were randomized to either receive 12-hourly subcutaneous injections of recIL-37 (40 µg/kg) or vehicle, then subjected to the same model as in **a**–**h**. Intestinal sections were obtained at the experimental endpoint and scored as above. Data are from three independent experiments; *n* = 12 for NEC + vehicle, 9 for NEC + recIL-37; Mann–Whitney *U* test *P*-value: *P < 0.05 for recIL-37 vs vehicle.

Just 10% of IL-37tg mouse pups died by the experimental endpoint at 72 h, whereas mortality was sixfold higher in WT pups (63%; i.e., mortality was 84% lower in IL-37tg pups, Fig. 2a). Mice that died from NEC exhibited reduced weight gain (Fig. 2b). Luminal gas trapping may be indicative of ileus, and gas within the intestinal wall is termed pneumatosis intestinalis, two pathologies observed in human NEC (Fig. 2c). Macroscopic assessment of the small intestine of the mouse pups revealed numerous gas bubbles in the intestinal lumen and within the intestinal wall in WT NEC mice, whereas IL-37tg NEC mice were largely protected from ileus and pneumatosis (Fig. 2d). Using a 0–3 scale (no to severe pathology, see “Methods”), three pathologies of human NEC exhibited lower scores in IL-37tg NEC mice than in controls: hematochezia by 88%, ileus by 79%, and, albeit non-significantly, diarrhea by 67% (Fig. 2e). Again based on a 0–3 scale (e.g., assessing vacuolation, tissue edema, mucosal disintegration; see “Methods” section and Supplementary Fig. 1), in the presence of IL-37, intestinal tissue damage was significantly reduced by 53% in the jejunum, whereas the 46% and 47% reductions in duodenum and ileum did not reach significance (Fig. 2f, g). Moreover, as indicated by the Shannon diversity index^25^, α-diversity of the microbiome (as reflected by even distribution and abundance of intestinal microbial species; taxonomies in Supplementary Fig. 2a) was preserved in IL-37tg pups exposed to NEC compared with WT NEC pups (Fig. 2h).

Employing a more translational approach, we next assessed the protection afforded by recombinant IL-37 (recIL-37)^26^ in this model. Although unsurprisingly at lower efficacy than transgenic expression, recIL-37 ameliorated NEC-associated injury in the jejunum by 38% (Fig. 2i). Other, non-significant observations included a reduction in duodenal disease severity (28%, Fig. 2i) and hematochezia (59%, Supplementary Fig. 2b), and there was no difference between recIL-37 and vehicle in terms of severity score in the ileum (Fig. 2i), ileus (Supplementary Fig. 2b) and survival (Supplementary Fig. 2c). We estimate that overall, recIL-37’s efficacy to protect from NEC was approximately half that of the transgene, based on averaging effect sizes in all categories (Fig. 2 and Supplementary Fig. 2).

Thus, transgenic expression of IL-37 conferred protection against NEC-induced tissue injury and associated pathologies. While not as strong, treatment with recIL-37 conferred modest protection. Of note, our murine NEC model mimicked the human disease in several aspects of clinical presentation as well as macro- and histopathology.

### Molecular characterization of murine NEC and IL-37 effects

**Fig. 3:**
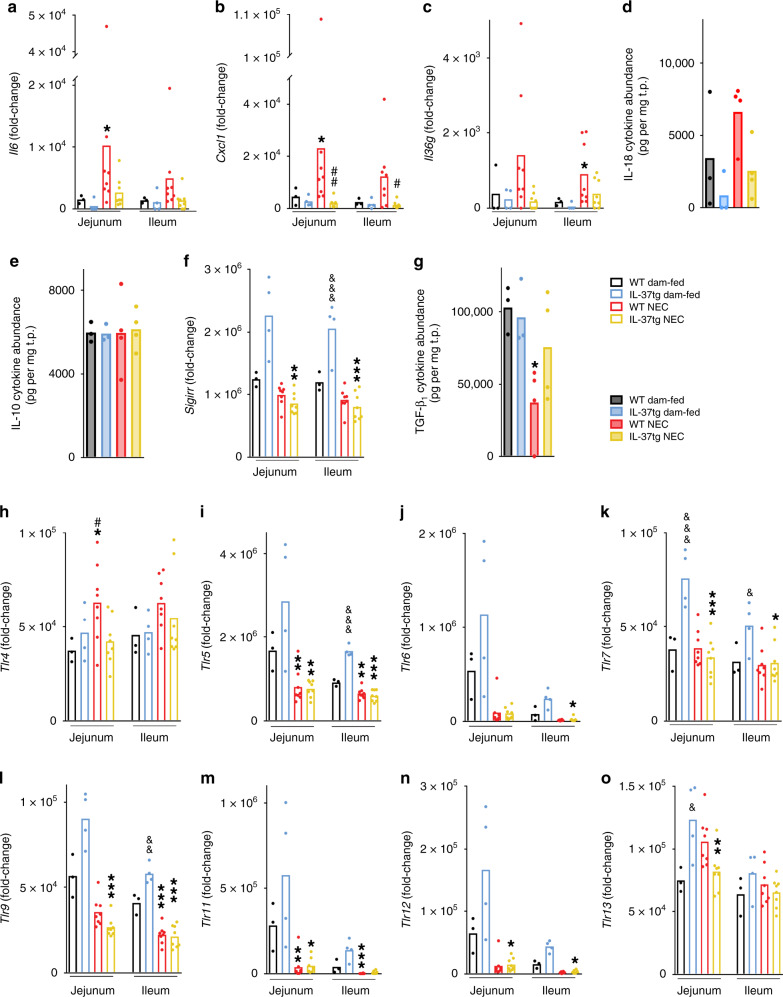
Pro- and anti-inflammatory mediators and TLRs in murine NEC and their modulation by IL-37. Intestinal tissue lysates from NEC or dam-fed pups collected at experimental endpoint or time of death were measured for gene expression (**a**–**c**, **f**, **h**–**o**, open bars) and protein abundance (**d**, **e**, **g**, filled bars) by multiplex real-time PCR and multiplex ELISA, respectively. Functionally, **a**–**d** are pro-inflammatory, **e**–**g** anti-inflammatory mediators, and **h**–**o** are TLRs. Data are from three independent experiments. Dots indicate data from individual mice and bars depict means. One-way ANOVA or ANOVA on ranks *P*-values: *P < 0.05; **P < 0.01; ***P < 0.001 for IL-37tg or WT NEC compared to dam-fed controls (see “Methods” section). ^#^*P* < 0.05; ^##^*P* < 0.01 for IL-37tg NEC compared to WT NEC. ^&^*P* < 0.05; ^&&^*P* < 0.01; ^&&&^*P* < 0.001 for IL-37tg dam-fed compared to WT dam-fed. **a**–**c**, **f**, **h**–**o** Real-time PCR results for the indicated genes were normalized to *Hprt1* and depicted as fold-change relative to the lowest expressed gene (see “Methods” section). *n* = 3 for WT dam-fed, 4 for IL-37tg dam-fed, 8 for both WT and IL-37tg NEC. **d**, **e**, **g** Ileal cytokine abundance of the indicated protein is normalized to total protein (see “Methods” section). *n* = 3 for both WT and IL-37tg dam-fed, 4 for both WT and IL-37tg NEC.

To explore the immunopathology of NEC, we subjected intestinal tissue lysates to multiplex real-time PCR and multiplex ELISA. In WT pups, NEC was associated with increased expression of pro-inflammatory genes in the jejunum and ileum compared to WT dam-fed mice (Fig. 1, box Mediators of inflammation), with up to sixfold increases in *Il6* (Fig. 3a), *Il1b* (Supplementary Fig. 3a), and C-X-C motif chemokine ligand 1 (*Cxcl1*; Fig. 3b). Similarly, of the three isoforms of the pro-inflammatory IL-1 family member IL-36, *Il36g* (Fig. 3c) was threefold increased, significantly so in the ileum, and *Il36a* and *b* (Supplementary Fig. 3b, c) were detectable only in the ileum of the NEC groups.

IL-37 prevented the development of the inflammatory milieu we observed in WT NEC pups: Expression of the pro-inflammatory genes *Il6*, *Il1b*, *Cxcl1*, and *Il36g*, but not *Il36b*, was similar to the dam-fed baseline in IL-37tg NEC mice (Fig. 3a-c, Supplementary Fig. 3a). As gene regulation was overall largely similar in jejunum and ileum, we proceeded to measure only ileal protein by multiplex ELISA. The NEC-associated twofold increase in IL-18 protein abundance in WT mice was prevented in IL-37tg NEC mice (*P* = 0.051; Fig. 3d). Similarly, the NEC-associated increase in TNF and IL-6 protein (Supplementary Fig. 3d, e) was significant in WT pups, but not in IL-37tg pups.

Next, we assessed the regulation of anti-inflammatory mediators by NEC and IL-37 in the intestine (Fig. 1, box Anti-inflammatory mediators). No change was observed across experimental groups for IL-10 (Fig. 3e), whereas *Il1rn* (protein name IL-1Ra) was 80% reduced by IL-37 in NEC pups (jejunum only, Supplementary Fig. 3f), and *Sigirr* (protein name IL-1R8) was twofold increased in IL-37tg at steady state but not in NEC (Fig. 3f). IL-36 receptor antagonist (IL-36Ra, gene name *Il36rn*) was undetectable in all but two mice (hence the IL-36 agonist:antagonist ratio could not be calculated in mice). Whilst changes in *Tgfb1* mRNA were minor (Supplementary Fig. 3g), ileal TGF-β_1_ protein abundance was 50% lower in WT NEC mice compared to WT dam-fed mice. This reduction in TGF-β_1_ was prevented in IL-37tg NEC mice (Fig. 3g).

These findings demonstrate that IL-37 counteracts NEC-induced inflammation in the murine small intestine in vivo. This protection was not via the anti-inflammatory cytokines IL-10 and IL-1Ra; however, IL-37 restored intestinal TGF-β_1_ to steady state.

### Changes in TLR abundance in murine NEC

Situated at the interface between host and microbiome at epithelial surfaces, TLRs are critical mediators of intestinal immune homeostasis^27^, and therefore a logical target for exploration in this study (Fig. 3h–o, Supplementary Fig. 3h–k and top two boxes in Fig. 1). Consistent with its known pathogenic role in NEC^28,29^, we observed an NEC-associated up to 1.7-fold increase in *Tlr4*, which was significant in the jejunum, and prevented by the IL-37 transgene (Fig. 3h). Our comprehensive study of mRNA regulation of TLRs furthermore revealed NEC-associated decreases in jejunal and ileal expression of *Tlr1* (up to −58%), *Tlr3* (−42%; Supplementary Fig. 3h, j), *Tlr5* (−74%), *Tlr6* (−93%), *Tlr9* (−70%), *Tlr11* (−94%), and *Tlr12* (−92%; Fig. 3i, j, l–n) in both WT and IL-37tg NEC pups compared to their respective dam-fed controls. Whereas the difference in *Tlr4* (Fig. 3h) and a decrease in jejunal *Tlr2* (−53%, Supplementary Fig. 3i) were the only significant changes conferred by IL-37 when we compared the two NEC groups, we unexpectedly found that the presence of IL-37 altered TLR expression in the intestines of control mice: There was an up to 3.4-fold increase in *Tlr5-7*, *Tlr9*, and *Tlr11-13* mRNA abundance (Fig. 3i–o, significant for *Tlr5*, *Tlr7*, *Tlr9*, and *Tlr13*) in IL-37tg compared to WT dam-fed mice. No significant change was observed for *Tlr8* (Supplementary Fig. 3k).

These data suggest that modulation of the intestinal baseline TLR repertoire may be one of the mechanisms IL-37 employs to confer its beneficial effects in NEC, highlighting the importance of this repertoire in intestinal health in the newborn. IL-37 also ameliorated the NEC-induced increase in TLR4.

### NEC-induced disruption of intestinal ILC homeostasis

**Fig. 4:**
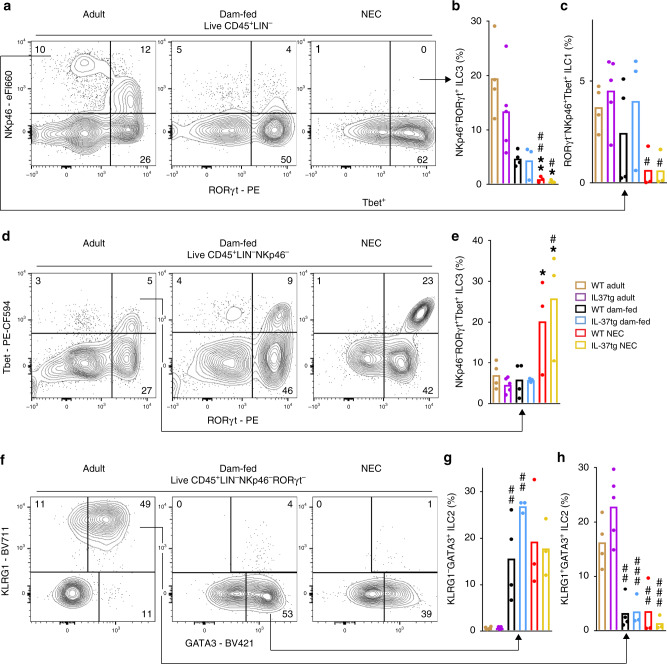
Developmental and NEC-associated changes in intestinal ILC subtypes. Lamina propria cells were isolated from the small intestine of 3 day-old pups (*n* = 4 for WT dam-fed and 3 each for IL-37tg dam-fed, WT and IL-37tg NEC from 3 independent experiments) and adult mice (*n* = 4 for WT and 5 for IL-37tg from 2 independent experiments) before being subjected to flow cytometric analysis. Cells were stained for NKp46, RORγt, and Tbet to identify ILC1 (**a**, **c**) and ILC3 (**a**, **b**, **d**, **e**) and for KLRG1 and GATA3 to assess ILC2 (**f**–**h**). **a**, **d**, **f** Plots show one representative result per group; arrows indicate the source quadrants of the percentage graphs, which are calculated as a percentage under total live CD45^+^ cells. **b**, **c**, **e**, **g**, **h** Percentage of NKp46^+^RORγt^+^ ILC3 (**b**), RORγt^−^NKp46^+^Tbet^+^ ILC1 (**c**), NKp46^−^RORγt^+^Tbet^+^ ILC3 (**e**), KLRG1^+^GATA3^+^ ILC2 (**g**), and KLRG1^−^GATA3^+^ ILC2 (**h**) is shown as data from individual mice (dots) and means (bars). One-way ANOVA or ANOVA on ranks *P*-values (for details, see “Methods” section): *P < 0.05; **P < 0.01 for IL-37tg or WT NEC compared to dam-fed controls. ^#^*P* < 0.05; ^##^*P* < 0.01; ^###^*P* < 0.001 for IL-37tg or WT NEC or dam-fed compared to adult mice. ^&^*P* < 0.05 for adult IL-37tg compared to adult WT.

The evidence for a notable relevance of ILC in intestinal disease is increasing^16,17^, but knowledge on ILC in NEC and in development during early life is scarce. Hence, we next investigated the developmental and NEC-associated changes to ILC populations in the murine small intestine (gating strategies in Supplementary Fig. 4a, group Innate lymphoid cells in Fig. 1). NKp46^+^RORγt^+^ ILC3, which have protective functions^30^, were almost completely ablated in NEC mice compared to adult and dam-fed mice (Fig. 4a, b); moreover, this ILC3 subset was up to 78% reduced in 3 day-old dam-fed pups compared to 8–10 week-old adult mice (Fig. 4b). Although ILC1 were also up to 85% lower in the NEC groups (Fig. 4a, c), the pro-inflammatory NKp46^−^RORγt^+^Tbet^+^ ILC3^31^ population was fivefold higher in NEC mice compared to dam-fed and adult controls (Fig. 4d, e). There was no difference in the abundance of pro-inflammatory ILC3 between adults and dam-fed controls (Fig. 4e), and only minor differences in NKp46^−^RORγt^+^Tbet^−^ ILC3 (Supplementary Fig. 4b).

As expected^32^, the majority of adult intestinal GATA3^+^ ILC2 exhibited the maturational marker KLRG1 whilst neonatal GATA3^+^ ILC2 were nearly devoid of KLRG1 (Fig. 4f-h). However, whether or not the 3 day-old pups were subjected to NEC induction made little difference to KLRG1 surface expression (Fig. 4h). In fact, NEC was not associated with any significant changes in ILC2 compared to dam-fed control pups of the same age; however, GATA3 positivity was up to 1.7-fold higher in IL-37tg adult and dam-fed ILC2 compared to their WT counterparts (Fig. 4g, h). This increase in GATA3 in control mice was the only change afforded by IL-37 on ILC populations (Fig. 4a–h).

In summary, NEC was accompanied by a marked increase in pro-inflammatory NKp46^−^RORγt^+^Tbet^+^ ILC3, and NEC intestines contained fewer ILC1 and presumably protective NKp46^+^RORγt^+^ ILC3. We also found notable differences between 3 day-old and adult mice in NKp46^+^RORγt^+^ ILC3 and ILC2, but not ILC1 and NKp46^−^RORγt^+^Tbet^+^ ILC3.

### IL-37 prevents dysregulation of adaptive immunity in NEC

**Fig. 5:**
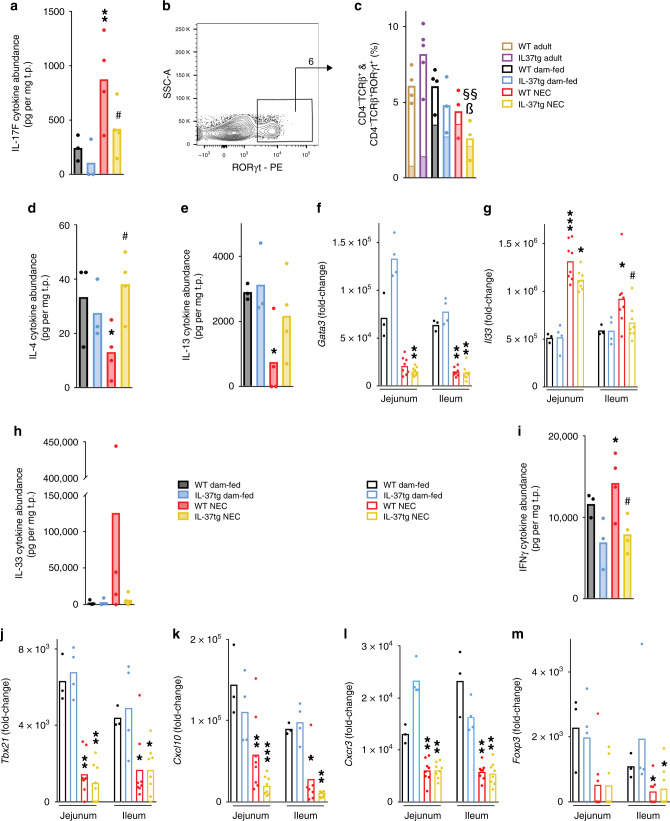
Markers of adaptive immunity in NEC. Intestinal tissue lysates were assayed for gene expression (open bars) by multiplex real-time PCR and protein abundance (filled bars) by multiplex ELISA or flow cytometry assessing mediators of adaptive immune type 3 (**a**–**c**), type 2 (**d**–**h**), type 1 (**i**–**l**) and regulatory (**m**) polarization. **a**, **d**–**m** One-way ANOVA or ANOVA on ranks *P*-values: *P < 0.05; **P < 0.01, and ***P < 0.001 for IL-37tg or WT NEC compared to dam-fed mice. ^#^*P* < 0.05 for IL-37tg NEC compared to WT NEC. ^&&&^*P* < 0.001 for IL-37tg dam-fed compared to WT dam-fed. **a**, **d**, **e**, **h**, **i** Ileal protein abundance of the indicated mediators is depicted as individual values (dots) and means (bars) normalized to total protein (t.p.). *n* = 3 pups for both WT and IL-37tg dam-fed, 4 for both WT and IL-37tg NEC. **b**, **c** Flow cytometric analysis for T cells on the same cells as in Fig. 4 was performed. As in Fig. 4, data are from *n* = 2–3 independent experiments; *n* = 4 mice for WT adult, 5 for IL-37tg adult, 4 for WT dam-fed, and 3 each for IL-37tg dam-fed, WT, and IL-37tg NEC. **b** Representative gating plot for CD4^−^TCRβ^+^RORγt^+^ cells, which originate from the live CD45^+^ lymphocyte gate; arrow indicates the source of the solid color fill in the graph. **c** Measurements from individual mice (dots) and means (open bars) of CD4^−^TCRβ^+^ cell percentages under live CD45^+^ lymphocytes are shown. One-way ANOVA *P*-values: ^§§^*P* < 0.01 for IL-37tg NEC compared to IL-37tg adult mice. ^ß^*P* < 0.05 for IL-37tg NEC compared to dam-fed (see “Methods” section). Solid color fill represents the mean percentage within each bar that is RORγt^+^, i.e., the RORγt^+^ fraction among CD4^−^TCRβ^+^ cells. Statistics for solid bars by one-way ANOVA (not shown in the figure): *P* < 0.001 for each of the groups of pups compared to adults. **f**, **g**, **j**–**m** Real-time PCR results were normalized to *Hprt1* and the indicated genes are graphed as fold-change relative to the lowest expressed gene (see “Methods” section). Dots indicate data from individual mice, bars indicate means. *n* = 3 mice for WT dam-fed, 4 for IL-37tg dam-fed, 8 for both WT and IL-37tg NEC.

We next investigated the regulation of adaptive immunity in NEC and its modulation by IL-37 (Fig. 1, encircled group Adaptive immunity). Consistent with the increase in the presumably pathogenic NKp46^−^RORγt^+^Tbet^+^ ILC3 population, NEC was associated with an increase in the protein abundance of T_H_17/type 3 cytokines, including IL-17F (fourfold, Fig. 5a) and IL-21 (19-fold though not statistically significant, Supplementary Fig. 5a) in WT pups. Furthermore, IL-22 was non-significantly higher (twofold, Supplementary Fig. 5b) and the *Il17a* and *Il22* genes were only detectable in jejunum and ileum of WT NEC pups (Supplementary Fig. 5c, d). However, there was little change in IL-17A and IL-23 protein abundance (Supplementary Fig. 5e, f), and the T_H_17/type 3 chemokine CCL20 was decreased in NEC (−44%, Supplementary Fig. 5g). The augmentation of the type 3 cytokines IL-17F and IL-21 in the NEC intestines was not observed in the presence of IL-37 (Fig. 5a and Supplementary Fig. 5a). Analysis of T cell numbers and polarization revealed that NEC was associated with an up to 68% decrease in intestinal CD4^−^ T cells (which are mostly CD8^+^ T cells; Fig. 5b, c); CD4^+^ T cells were also up to 91% decreased (Supplementary Fig. 5h, i). Importantly, among CD4^−^ T cells, the fraction that was T_H_17/type 3-polarized was sixfold increased (78% vs 13%; *P* < 0.001) in NEC compared to adults (Fig. 5c), and, albeit not significantly, the same was the case in CD4^+^ T cells (1.5-fold in WT NEC and threefold in IL-37tg NEC, Supplementary Fig. 5i).

The type 2 cytokine balance was also disrupted in WT NEC mice, with decreased abundance of IL-4 (−60%) and IL-13 (−74%; Fig. 5d, e). Likewise, the expression of *Gata3* (Fig. 5f) and protein abundance of CCL11 (Supplementary Fig. 5j) were reduced by 75% and 44%, respectively. Jejunal and ileal *Il4* and *Il13* gene expression were virtually undetectable in WT NEC mice (Supplementary Fig. 5k, l), and TSLP (thymic stromal lymphopoietin) was undetected except in one dam-fed mouse. Interestingly, mRNA and protein abundance of the epithelial type 2 cytokine IL-33 were up to 40-fold increased in NEC pups compared to their dam-fed littermates (Fig. 5g, h). IL-37 normalized the NEC-associated dysregulation of IL-4, IL-13, and IL-33 (Fig. 5d, e, g, h), restoring each to steady state, but did not affect CCL11 and *Gata3* (Supplementary Fig. 5j and Fig. 5f).

Investigation of markers of type 1 immunity yielded a mixed picture. Whereas we observed moderate increases in *Cxcl11* (Supplementary Fig. 5m, jejunum only) and IFNγ (Fig. 5i) in WT NEC mice compared to dam-fed controls, IL-12p70 protein was unchanged (Supplementary Fig. 5n). In contrast, we found the transcription factor Tbet (gene name *Tbx21*, Fig. 5j), the chemokine *Cxcl10,* and the chemokine receptor *Cxcr3* (Fig. 5k, l) reduced by up to 77%. Regulation of type 1 mediators by IL-37 was also not uniform. IFNγ abundance (Fig. 5i) trended lower in IL-37tg than in WT pups at steady state and in NEC, whereas there was no effect on *Tbx21*, *Cxcl11,* and IL-12 (Fig. 5j and Supplementary Fig. 5m, n). However, IL-37 reduced *Cxcl10* even further in NEC (Fig. 5k), but increased *Cxcr3* at steady state in the jejunum (Fig. 5l).

The transcription factor that predominates in regulatory T cells (Treg), *Foxp3*, was reduced by up to 77% in both WT and IL-37tg NEC mice compared to their respective dam-fed controls (Fig. 5m).

Overall, these findings indicate that a type 3 response in combination with a deficiency in type 2 cytokines and in Tregs may contribute to murine NEC. IL-37 largely restored the cytokine imbalance but did not affect the intestinal abundance of *Foxp3*.

### Characterization of the pathoimmunology of human NEC

**Fig. 6:**
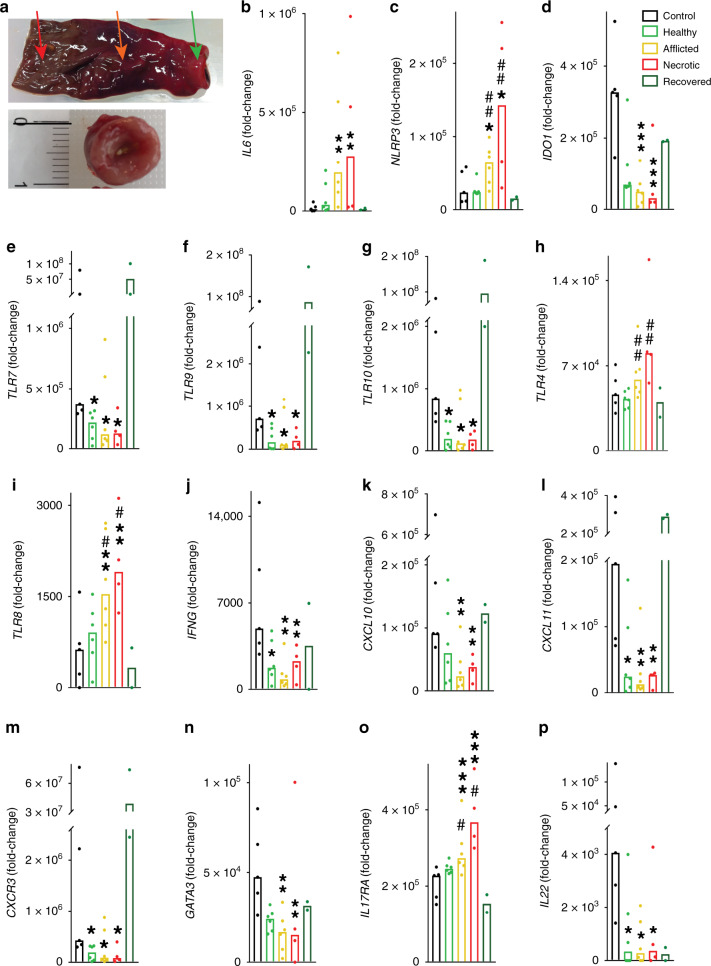
Innate and adaptive immunity in human NEC. Intestinal tissue sections were collected from infants with acute surgical NEC (*n* = 6 healthy/afflicted, *n* = 4 necrotic), from the same infants upon recovery from NEC (at reanastomosis of the stoma, *n* = 2), and from infants who underwent intestinal surgery for non-inflammatory diseases other than NEC (*n* = 5). For further clinical information, see Supplementary Table 1. The resection specimens were assessed for gene expression by multiplex real-time PCR. **a** Upper panel, acute NEC specimens were macroscopically divided into healthy (green arrow), afflicted (orange arrow) and necrotic (red arrow) sections; one exemplary resection specimen is shown. Lower panel, exemplary specimen at time of reanastomosis. Categories of mediators shown are: innate immunity (**b**–**i**); type 1 (**j**–**m**); type 2 (**n**); and type 3 (**o**, **p**) adaptive immunity. **b**–**p** Real-time PCR results were normalized to *ACTB* and graphed as fold-change relative to the lowest expressed gene. Measurements of individual infants are depicted as dots, bars are medians. One-way ANOVA on ranks *P*-values: *P < 0.05; **P < 0.01, and ***P < 0.001 for healthy, afflicted or necrotic acute NEC compared to non-NEC controls. ^#^*P* < 0.05 and ^##^*P* < 0.01 for healthy NEC vs afflicted or necrotic NEC (see “Methods” section).

As pathoimmunological phenotypes in mice not rarely diverge from those in humans (and NEC being notorious for such divergence^12^), a critical next step was to validate our mouse findings in samples from human infants with NEC. We investigated two separate cohorts and sample types: (1) surgical tissue resection specimens from human preterm infants with stage III, i.e., advanced NEC for which surgery is usually required^33^, at the time of surgery (exemplary specimen with macroscopically healthy, afflicted and necrotic intestinal regions indicated by arrows in Fig. 6a, upper panel) as well as at the time of reanastomosis (e.g., Fig. 6a, lower panel), and from appropriate controls (overview of clinical data in Supplementary Table 1). (2) longitudinal blood samples from infants with NEC stages I–III and pertinent controls.

Gene expression analysis on the intestinal samples from cohort 1 revealed that consistent with our murine NEC data (Fig. 3 and Supplementary Fig. 3), expression of pro-inflammatory genes in afflicted and necrotic human NEC sections was increased compared to each of the three types of controls (Fig. 6). These controls were (a), intestinal tissue from the same infant obtained at the same time as the afflicted and necrotic regions termed ‘Healthy’; (b) from the same infant after recovery from NEC termed “Recovered”; and (c) from non-inflamed intestines termed “Control” (see “Methods” section for details on control infants). The increased genes included *IL1B* (up to 116-fold, Supplementary Fig. 5a), *IL6* (28-fold, Fig. 6b), *TNF* (42-fold, Supplementary Fig. 5b), *IL8* (289-fold, Supplementary Fig. 5c), and *IL1A* (43-fold, Supplementary Fig. 5d; changes in supplementary figure did not reach statistical significance due to intra-group variability). We also observed a trend towards increased expression of the IL-36 isoforms *IL36A* (mostly undetectable in each of the healthy groups, Supplementary Fig. 5e) and *IL36B* (mostly undetectable in Control and Recovered, but the higher expression in Healthy, Supplementary Fig. 5f) and *IL36G* (up to 2.5-fold, Supplementary Fig. 5g) in “Afflicted” and “Necrotic” NEC sections compared to controls. These findings also resembled those in murine NEC (Fig. 3 and Supplementary Fig. 3). Although *IL36* gene expression was relatively low, the ratios between the pro-inflammatory IL-36 agonists and IL-36RA were substantially higher in the NEC groups (up to 102-fold for *IL36B*:*IL36RN* and up to 50-fold for *IL36G*:*IL36RN*). In addition, we found expression of the inflammasome component *NLRP3* up to ninefold increased (Fig. 6c). In the majority of cases, there was no or little difference in the expression of pro-inflammatory genes between non-inflammatory Controls, the macroscopically Healthy regions during acute NEC, and intestines of infants who had Recovered from NEC (Fig. 6b and Supplementary Fig. 6).

Regarding anti-inflammatory/regulatory mediators, there was little difference in *TGFB1* expression between the groups (Supplementary Fig. 6i), but *IL10* was increased more than fourfold in afflicted and necrotic NEC sections (Supplementary Fig. 6j). In contrast, *IDO1* was reduced by 90% (Fig. 6d). See above for *IL36RN* and below for the IL-37 pathway.

TLR expression in human NEC was mostly bipolar, with only *TLR1* and *TLR3* showing little change (Supplementary Fig. 7a, b). Whilst *TLR7, TLR9*, *TLR10* (Fig. 6e–g), *TLR2*, *TLR5*, and *TLR6* (Supplementary Fig. 7c–e) were markedly decreased by up to 86% in each of the regions of the acute NEC specimens, *TLR4* (twofold, Fig. 6h) and *TLR8* (sixfold, Fig. 6i) were increased particularly in the necrotic NEC sections compared to the Control and Recovered groups.

Type 1 mediators declined more consistently in humans than murine NEC: *IFNG*, *CXCL10*, *CXCL11,* and *CXCR3* (Fig. 6j–m), as well as *TBX21* (Supplementary Fig. 7f), were up to 93% reduced in acute NEC specimens compared to non-inflammatory Controls. In terms of type 2 cytokines, the pathoimmunology of human and mouse NEC was largely congruent, although due to intra-group variability, most differences did not reach statistical significance. This was the case for the NEC-associated reductions in *IL5* and *IL13* (Supplementary Fig. 7g, h), whereas the 68% decrease in *GATA3* was significant (Fig. 6n). Intriguingly, even the NEC-associated increase in *IL33* we observed in murine NEC was present as a twofold increase in human disease (Supplementary Fig. 7i).

Regarding type 3 and Treg mediators, we observed an NEC-associated twofold increase in the gene encoding a receptor subunit of IL-17A and F, *IL17RA* (Fig. 6o), whereas *IL22*, which is generally considered protective in the intestine, was up to 93% lower (Fig. 6p). NEC did not affect the expression of *IL17A* and *RORC* (Supplementary Fig. 7j, k), but in contrast to our murine findings, *FOXP3*, *IL17F* and *IL21* trended lower in each of the intestinal regions of infants with acute NEC (Supplementary Fig. 7l–n).

**Fig. 7:**
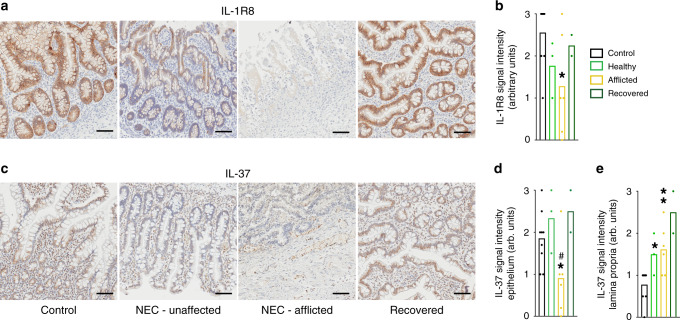
Intestinal IL-37 and IL-1R8 in human NEC infants. Immunohistochemical assessment of IL-1R8 (**a**, **b**) and IL-37 (**c**–**e**) in samples from the same infants, namely non-NEC controls (control, *n* = 9), largely healthy NEC tissue (unaffected, *n* = 3), NEC-afflicted tissue (afflicted, *n* = 6) and samples from intestines after NEC recovery (recovered, *n* = 2). “Afflicted” also comprises the necrotic group in the immunohistochemistry experiments. **a**, **c** One representative image for each group is shown. Scale bars: 50 µm. **b**, **d**, **e** Quantification of the signal intensity of IL-1R8 (**b**) or IL-37 in the epithelium (**d**) or the lamina propria (**e**). Bars plotted indicate means of individual signal intensity values, which are shown as dots. One-way ANOVA *P*-values: *P < 0.05 and **P < 0.01 for healthy or afflicted acute NEC compared to non-NEC controls. ^#^*P* < 0.05 for healthy vs afflicted sections in acute NEC.

One of our most intriguing observations was that the IL-37 pathway is compromised in NEC. By immunohistochemistry (Fig. 7a–e), IL-1R8 (previously called SIGIRR or TIR8) was readily detectable in epithelial cells in the villi and crypts of non-NEC Controls and infants Recovered from NEC (Fig. 7a, far left and far right, Fig. 7b), whereas intact villi in NEC tissue exhibited a reduced IL-1R8 abundance (Fig. 7a, 2nd from left, Fig. 7b). IL-1R8 was almost completely absent in regions where the integrity of the villi was compromised (Fig. 7a, 3rd from left). Quantitatively, IL-1R8 fell by 43% in the NEC-afflicted regions and by 31% in the non-afflicted/healthy regions (Fig. 7b). Of note, there was virtually no IL-1R8 staining in the tissue directly underlying the epithelium, termed “lamina propria” (Fig. 7a). In contrast, IL-37 was detectable in each of the layers of the intestinal wall we investigated (Fig. 7c), and interestingly, its abundance was differentially affected by NEC: On the one hand, epithelial IL-37 was present in most cells of non-NEC controls and recovered NEC tissue (Fig. 7c, far left and far right, Fig. 7d), whereas epithelial IL-37 was decreased by 51% in NEC-afflicted tissue (Fig. 7c, 3rd from left, Fig. 7d). On the other hand, IL-37 abundance was moderate in the lamina propria of the Control group, whereas we found twofold increased IL-37 in the NEC intestines, with most of the staining localized to the infiltrating immune cells (Fig. 7c, e, monocytes in Fig. 1).

**Fig. 8:**
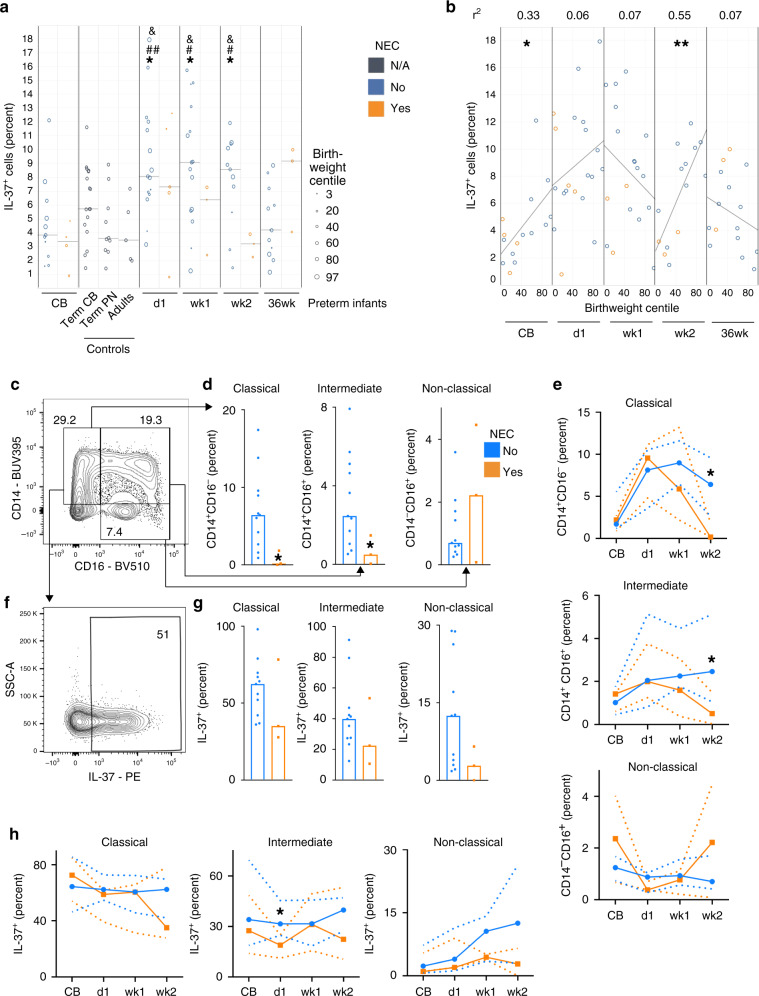
IL-37 abundance in blood cells from human NEC infants. Peripheral blood was obtained from the second cohort of premature infants (gestational age 24–29 weeks, *n* = 21; note that some time points are not available for some preterm infants; for exact *n* at each time point see dots in panels and Source Data file) at the indicated time points, from healthy term infants at birth (*n* = 17) and at 4–16 weeks of age (*n* = 10), as well as from healthy adult volunteers (*n* = 5). The percentage of IL-37^+^ leukocytes among viable CD45^+^ cells was determined by flow cytometry. CB cord blood, Term healthy term infants, PN postnatal, d1 day 1, wk1/2 week 1/2, 36wk 36 weeks of corrected gestational age. **a**, **b** IL-37^+^ percentages are graphed for each individual infant (circles) with medians indicated by gray lines. Student’s *t*-test *P-*values: *P < 0.05 for indicated preterm time point vs preterm CB. ^#^*P* < 0.05 and ^##^*P* < 0.01 for indicated preterm time point vs term PN. ^&^*P* < 0.05 for indicated preterm time point vs adults. **b** Correlation of birthweight centile with IL-37^+^ percentage at the indicated time points. Pearson correlation *P-*values: *P < 0.05; **P < 0.01. **c**–**h** Analysis of monocyte subtypes and their IL-37^+^ percentages at the week 2 time point (**c**, **d**, **f**, **g**) or longitudinally (**e**, **h**). Mann–Whitney *U* test *P-*values: *P < 0.05 for NEC vs non-NEC. **c**–**e** Quantification of monocyte subtypes under viable CD45^+^ cells, and **f**–**h** of IL-37^+^ percentages under the indicated viable CD45^+^ monocyte subtypes; **c**, **f** Exemplary plots of gating strategies, with percentages indicated by numbers within or next to boxed fields. **d**, **g** Quantification of individual infants shown by dots, bars denote medians. **e**, **h** Median values across the indicated time points are shown by solid lines, dotted lines are interquartile ranges (IQRs).

To further explore IL-37 in human NEC with a particular focus on immune cells by flow cytometry (gating in Supplementary Fig. 8), we analyzed blood samples from a separate second cohort of 21 infants born between 24 and 29 weeks of gestational age (patient characteristics in Supplementary Table 2), from whom blood was taken at birth, on day 1, in weeks 1 and 2 and at 36 weeks corrected gestational age; 27 healthy term infants (17 sampled at birth, 10 at 4−16 weeks of age) and 5 adults served as controls. In healthy term infants, median IL-37 abundance among CD45^+^ cells at birth was 5.7% and decreased to 3.6% within the first months of life, which was almost identical to the adult percentage (3.7%, Fig. 8a). In preterm infants who did not suffer from NEC, IL-37 was somewhat lower at birth (median 3.8%), then increased more than twofold to up to 9.1% at week 1, before returning to a baseline of 4.2% at 12–16 weeks of life. In infants who went on to develop NEC, we also observed a twofold increase from cord blood to day 1, but thereafter median IL-37 abundance fell to a nadir of 3.2% at week 2, which aligns with the age at which NEC occurs most frequently (Fig. 8a). In other words, the longitudinal increase in circulating IL-37^+^ cells that occurs in non-NEC infants in the first 2 weeks of life is less pronounced in NEC infants, particularly in week 2. As intrauterine growth restriction (IUGR) is an established risk factor for NEC, we also plotted IL-37 against the infants’ birth weight centiles, revealing that IUGR correlated with lower IL-37 abundance at birth and in week 2 (Fig. 8b).

Deeper cell-type analysis of the cohort 2 data revealed NEC-associated reductions in classical and intermediate monocyte populations, but not in non-classical monocytes, which trended higher in NEC (Fig. 8c–e). In contrast, IL-37^+^ cell percentages were lower in all three monocyte subgroups in NEC infants from day 1 onwards (Fig. 8f–h). In line with Fig. 8a, differences were most striking in week 2, when classical and intermediate monocytes were almost completely lost (−97%, Fig. 8d, e), and IL-37^+^ percentages were up to 78% lower in remaining monocytes (Fig. 8g, h). Except in intermediate monocytes on day 1, the differences in IL-37 abundance were less pronounced or absent at the other time points (Fig. 8h). IL-37^+^ percentages were low throughout in T cells (combined non-NEC and NEC median, 0.34% ± IQR 0.2–0.7%), B cells (3.1% ± 2–6.5%), NK cells (3.8% ± 1.3–16.7%) and neutrophils (1.3% ± 0.5–3%), which were therefore not analyzed further.

Overall, our investigations of human samples reveal that NEC is associated with a deficiency in IL-37 (systemic and epithelial, but not subepithelial) and its receptor IL-1R8 (epithelial). Moreover, our human findings are largely in accordance with our murine data by corroborating the NEC-associated imbalance in pro- and anti-inflammatory cytokines, adaptive immunity, and TLRs. However, as expected, we also observed some discrepancies, for example in the regulation of CCL11 and CXCL11. Figure 1 provides an overview of our human and murine findings.

## Discussion

Despite decades of research, NEC remains a major challenge in the neonatal intensive care unit because of its insidious onset and rapid progression and the absence of an effective therapy, which renders neonatologists powerless to treat what still is for many infants a deadly disease and for survivors a severely disabling condition. As the main obstacle to developing new treatments has been our poor pathomechanistic understanding, we set out to parse the destructive inflammation underpinning NEC. Several discoveries emerge from our work: (i) the wide-spectrum anti-inflammatory cytokine IL-37 and its receptor IL-1R8 are deficient in human NEC, and augmenting IL-37 ameliorates the disease in mice. (ii) Beyond the promise these findings hold for future use of IL-37 to treat or prevent NEC, our exploration of the beneficial effects of IL-37 yields mechanistic insights, including (iii) an unexpected IL-37-mediated modulation of baseline TLR expression, and (iv) partial restoration by IL-37 of the imbalance in type 3 vs type 1 and 2 adaptive immunity as well as in innate pro- and anti-inflammatory mediators. (v) Type 3 immunity unexpectedly dominates over types 1, 2 and Treg in NEC, as reflected in an imbalance of ILC, T cells and mediators such as IL-17 and its receptor, IL-22 and GATA3; (vi) both human and murine NEC exhibit complex TLR dysregulation with pronounced decreases in TLRs 5–7 and 9-12 and increases in TLRs 4 and 8; and (vii) the disease is associated with abnormalities in the IL-36 pathway. These findings constitute a substantial advance on the path to understanding and future conquest of NEC.

Innate immunity matures faster than adaptive immunity during gestation^34^, and pregnancy skews the fetal adaptive immune system towards type 2 polarization^35^. Our finding that type 3 mediators may contribute to NEC is thus unexpected. One report linked intestinal IL-17A (IL-17F was not measured) and IL-17RA to murine and human NEC;^36^ in that study, blockade of IL-17RC, a receptor subunit for IL-17A&F, abrogated murine NEC injury^36^. A pathogenic role for T_H_17 signaling in NEC would be consistent with increases in IL-17A, IL-17F, and T_H_17 cells in ulcerative colitis (UC) and Crohn’s disease (CD)^37,38^—and the signaling pathways affected in CD and NEC are not dissimilar^39^. The NEC-associated increase in IL-17F we observed corroborates human genetic evidence that an SNP in *IL17F* renders infants 2–3 times as likely to suffer from advanced NEC^40,41^.

In the intestine, IL-22 generally exerts protective functions. The NEC-associated decrease we observed in *IL22* in human NEC and in murine RORγt^+^NKp46^+^ ILC3, which are prominent IL-22-producers^31^, indicates a possible involvement in NEC. However, IL-22 function is dependent on the cytokine milieu^42^, and is thus not straightforward to dissect in the cytokine storm of NEC. Overall, our data on type 3 cytokines suggest that manipulation of the IL-17 and/or IL-22 pathways could bestow therapeutic benefit.

In addition to type 3 cytokine dysregulation, we reveal an association between mouse NEC and an increase in pro-inflammatory NKp46^−^RORγt^+^Tbet^+^ ILC3, alongside a reduction in presumably protective NKp46^+^RORγt^+^ ILC3^30^, corroborating a protective role for this ILC3 subtype, as increased IL-17A^+^IL-22^+^ ILC3 populations were reported in P.UF1-gavaged mice that were protected against NEC^43^. Thus, we highlight an imbalance of ILC3 subtypes in NEC and substantially expand on a previous report on ILC3-mediated, though not NEC-related, intestinal pathology in neonatal mice^17^. Whilst ILC3 generally feature a type 3 effector cytokine profile (analogous to T_H_17 cells), the presence of Tbet in NKp46^−^RORγt^+^ ILC3 identifies a subset that produces pro-inflammatory cytokines^31^. A similar switch occurs in T_H_17-polarized T cells, which then co-express Tbet and RORγt and promote chronic inflammation^44^. In fact, whilst we observe decreases in CD4^+^ and CD4^−^ T cells, the remaining T cells exhibit an increase in type 3 polarization, and a disease-aggravating role for T cells would be consistent with a study showing that Rag-deficient mice are protected from NEC^36^. Further, the NEC-associated expansion of the pro-inflammatory NKp46^−^RORγt^+^Tbet^+^ ILC3 population may represent a response to compromised epithelial integrity and subsequent bacterial invasion^31^. Whatever the cause, the expansion of pro-inflammatory ILC3 corroborates our observation of the NEC-associated increase of their disease-promoting effector cytokines IFNγ and TNF^45,46^.

Next, we found an association of NEC and decreased intestinal abundance of type 2 mediators such as IL-13 and GATA3 in human and murine NEC specimens. This finding was unexpected given the fetal and neonatal bias towards type 2 polarization in mice^47^ and humans^35^, and stands in contrast with a report on increased ileal IL-4, IL-5, and IL-13 in NEC rats^48^, which may be due to differences in study design. Pathoimmunologically, such weakening of type 2 pathways places NEC closer to CD and remote from UC, where type 2 immunity is usually considered pathogenic^49^. Accordingly, our data agree with reports of IL-13 being protective in chronic models of murine colitis^50^, and increased *Il4* in pioglitazone-mediated protection against NEC^51^. The NEC-associated intestinal deficiency in type 2 mediators may in part be due to the age-dependent lack of KLRG1^+^ ILC2 in neonatal mice we found, as KLRG1 was identified as a marker of type 2 cytokine-producing intestinal ILC^32^.

The combination of the dominance of type 3 mediators and weakening of types 1, 2, and Treg in NEC points to a profound imbalance within the adaptive immune system. One function of type 2 cytokines is to hold type 3 responses in check; examples studied in neonatal models include IL-4^52^ and IL-13^53,54^. The NEC-associated reduction in parameters of type 1 immunity, including ILC1 in mice and IFNγ and type 1 chemokines in human infants to our knowledge has not been shown before. Our data on reduced FoxP3 expression in NEC are in accord with lower Treg abundance reported by others^36^.

Turning to innate immunity and inflammation, our results align well with previous literature in showing the intestinal inflammation of NEC to be characterized by augmented IL-6 and TNF, as well as reduced TGF-β and TLR9, as we reviewed in ref. ^12^. Beyond this, we reveal a novel link between NEC and IL-36 cytokines, including NEC-associated rises in agonist:antagonist ratios for IL-36β and IL-36γ. These findings agree with reports on increased IL-36α and γ in UC and CD in humans^55–57^, but knowledge on IL-36 function in the intestine is sparse overall. There are conflicting data on IL-36R signaling in intestinal inflammation, e.g., in acute DSS-colitis^56,58^ and chronic DSS/TNBS-colitis^59^. However, mice deficient in IL-36γ are protected from T_H_ cell-driven intestinal inflammation, and IL-36γ inhibited Treg development^60^, suggesting a disease-augmenting role for IL-36 in the gut.

The repertoire of the ten human (termed TLR1-10) and 12 murine (TLR1-9, TLR11-13) TLRs on intestinal epithelial (IEC) and immune cells is of critical importance in determining whether signals from commensal bacteria result in tolerance or lead to perturbed homeostasis, inflammation, and tissue injury^61^. Past NEC research focused mostly on TLR4^28,29^ and TLR9^62^, for which disease-aggravating and -ameliorating roles are reported, respectively. We confirm a NEC-associated increase in TLR4 and decrease in TLR9 in human and mouse. Consistent with TLR5-deficient mice developing spontaneous colitis^63^, we demonstrate that the potentially protective TLR5^48^ is decreased in rodent and human NEC. For TLRs 1–3, we did not observe the previously reported augmented expression in NEC^48^; instead, we found inconsistent regulation. Moreover, we observed a decline in the relatively understudied TLRs 11 and 12, neither of which has been investigated in NEC to our knowledge. TLR10 is reduced in human NEC, possibly supporting emerging evidence for its anti-inflammatory function^64^. Thus, our work consolidates the reported regulation of TLRs 4 and 9 in NEC, adds to the previously scant knowledge on TLRs 5 and 6, and sheds first light on TLRs 8 and 10–13.

IL-37 is a powerful, broadly-acting anti-inflammatory member of the IL-1 cytokine family, whose function^21^, cell-surface receptor IL-18Rα:IL-1R8 and signaling^26,65^, we have described. Because its protective properties cover intestinal diseases^23,66–68^, we investigated IL-37 in NEC. Besides establishing IL-37 as a potential therapeutic approach in NEC, this aspect of our data provides pivotal mechanistic insights by demonstrating which pathways are involved in IL-37’s rescue from the disease and which are not.

Consistent with the known functions of IL-37^21,22,65,69–72^, IL-37tg pups were nearly completely protected from the NEC-driven increases in IL-1β, IL-6, TNF, and IL-17F, and from the reduction in TGF-β_1_. Contrary to expectations, IL-37 had little effect on type 1 mediators such as IL-12 and IL-23, the distribution of ILC subsets, nor on IL-10 in NEC. Another intriguing finding was that baseline TLR repertoires differed markedly between IL-37tg and WT pups, with increased abundance particularly in the protective TLRs 5^63^ and 9^62^, and also in TLRs 6, 7, and 11–13. The fact that IL-37tg pups were protected from loss of intestinal microbial diversity, a prominent feature of NEC^10,73^, indicates that dominance of TLRs 5–7, 9, and 11–13 may offer a degree of protection from the pathological signals triggered by NEC-associated alterations in the microbiome.

The IL-37-induced baseline increase in IL-1R8 likely further augments this inherent protectiveness of the intestinal milieu in IL-37tg, given that loss-of-function mutations in *IL1R8* (gene also called *SIGIRR*) are associated with NEC^74^, and that IL-1R8 is a negative regulator of IL-1R- and TLR-dependent inflammation^65,75^. Indeed, IL-1R8-deficiency leads to TLR4 hyper-responsiveness and more severe intestinal inflammation and tissue injury in NEC models^76^. The localization of IL-1R8 to IEC of neonatal mice^76^ is consistent with our findings in human preterm neonates. The high abundance of IL-1R8 in IEC at steady state suggests that these cells are the primary targets for extracellular and luminal IL-37 in the human neonatal intestine. IL-37 itself was detectable in IEC and in interstitial cells at steady state; in NEC, however, epithelial IL-37 declined, whereas lamina propria IL-37 increased. While as expected^21,77^, infiltrating leukocytes were the main source of this increase, these data in their entirety suggest that the contribution of epithelial IL-37 and IL-1R8 is most critical to maintaining immune homeostasis and rendering the environment tolerogenic to signals from the commensal microflora.

Our observation that the protective capacity of postnatal injection of IL-37—which in our 3 day-model cannot instantaneously augment its own receptor or alter the intestinal TLR repertoire—is less pronounced than that of the transgene supports the hypothesis that modulation of TLR repertoire and microbiome plays an important part in the protective properties of the IL-37 pathway. However, it may be possible to improve the efficacy of recIL-37 by optimizing dosage and timing in future mouse experiments.

In human preterm infants, NEC most commonly occurs around d14 of life or later^78^—which aligns remarkably well with a fall in classical and intermediate monocytes, the main sources of IL-37 among blood cells^77^, in our second cohort. While such falls in monocyte counts have been associated with NEC onset before^79,80^, we moreover observed that only the prototypically anti-inflammatory monocyte subtypes^81^ are affected. Intriguingly, the NEC-associated depletion of these monocytes was further compounded by a decrease in IL-37 in the remaining monocytes, resulting in a profoundly depleted pool of circulating IL-37. It has been proposed that the severe inflammation of NEC leads to an exodus of monocytes into the intestine, which is consistent with macrophage-rich infiltrates in tissue specimens^82^. As monocytes carry 80–90% of blood-borne IL-37^77^, the increase in lamina propria IL-37 we observed may be due to the infiltration of IL-37^+^ monocytes, as part of the body’s attempts to curtail runaway inflammation. From the translational perspective, we conclude that IL-37-based therapies should commence early, possibly even in a preventative fashion, particularly when risk factors such as IUGR are present. To optimize the benefits of the mucosal microenvironment, oral administration should be considered.

Taken together, our observations strongly suggest a deficiency in IL-37 and IL-1R8 contributes to NEC pathogenesis. As NEC advances, cytokine and receptor disappear from the epithelium, and IL-37^+^ percentages fall among systemic monocyte subsets, thus likely accelerating the vicious cycle of excessive inflammation and intestinal injury^12^. Transgenic IL-37 protected mouse pups by restoring homeostasis in selected pathways of innate and adaptive immunity and in the microbiome. As a next translational step towards IL-37-based therapies, dosage, timing, and route of administration should be examined.

As a substantial part of the value of our novel findings hinges on the fidelity of the mouse NEC model, it is important to highlight the high degree of conformity between clinical pathologies, the pattern of tissue injury, and molecular data obtained in murine and human neonates. Despite the relatively small number of human participants, many (though not all) major pathways and mediators exhibited congruent regulation, suggesting that our findings in the mouse are largely translatable to the human.

In conclusion, our work represents a compendious addition to current knowledge of the pathomechanisms that drive NEC. Our focus on the largely uncharted aspect of adaptive immunity reveals a predominance of type 3 polarization over types 1 and 2, including a role for T_H_17 T cells and a pro-inflammatory ILC3 subtype. IL-37 afforded powerful protection from NEC, at least in part by restoring a balanced type 3/2 polarity, and by modulating TLR expression, microbial homeostasis, and the IL-36 pathway. Our study thus points to promising therapeutic strategies among these cascades and mediators, such as blocking IL-17F, IL-36, and TLR4, or augmenting TLR5 and 9. Although the impact of TLRs 6–8 and 10–13 in NEC remains to be proven, our data suggest they are implicated in this disease. Perhaps the finding with the greatest translational promise is that boosting the IL-37 pathway, which we discovered to be deficient in human NEC, confers almost complete protection in the mouse. However, more work is required to optimize the efficacy of exogenously administered IL-37. We speculate therefore that IL-37 in particular, but other strategies identified here as well, could provide our tiniest patients with a much-needed therapy to shield them from the ever-looming specter of NEC.

## Methods

### Animal model

All animal work was approved by Monash Medical Centre Ethics Committee A (MMCA; approval numbers MMCA/2012/62 and MMCA/2017/30) and was conducted in accordance with the principles of the Declaration of Helsinki. C57BL/6J wild-type (WT) mice were originally purchased from Jackson Laboratories (USA; stock number 000664) and the colony was maintained by the Monash Animal Research Platform. IL-37 transgenic (IL-37tg) mice were homozygous offspring from the original colonies^21^. IL-37tg and WT animals for experimental and control groups were bred in the same room under enhanced specific pathogen-free (SPF)2 conditions; experiments were performed under conventional conditions. A 12 h/12 h dark/light cycle was maintained throughout experimental and breeding settings. Only mice between 8 weeks and 12 weeks were used as breeders. All dams were time-mated so that wild-type and IL-37tg pups could be evaluated in parallel. Half the mice of each wild-type and IL-37tg litter were separated from their dams within ~6 h after birth. The other half of each litter remained with their dams to serve as time-matched dam-fed controls. The pups separated from the dams were co-housed in a human neonatal incubator at 33–34 °C and 60–70% humidity. Based on a published murine model of NEC^83^, pups were hand-fed low lactose formula milk (PEDIGREE Puppy Milk, McLean, USA) every 3 h for 72 h using the Hoshiba-Yajima style nipple (Meiji Dairies, Japan)^84,85^. In addition, these pups were stressed every 12 h with asphyxia (100% nitrogen gas for 60 s) followed 5 min later by cold stress (placement in a refrigerator at 4 °C for 5 min). Pups were humanely killed by decapitation as per the guidelines of the NIH’s Animal Care and Use Committee (https://oacu.oir.nih.gov/animal-research-advisory-committee-guidelines) either at the 72 h experimental endpoint or when in distress (defined as little response to handling, severe weight loss, at least moderate abdominal distention, intra-abdominal bleeding or rapid, abdominal breathing, and recorded on animal monitoring sheets).

For recIL-37 experiments, newborn wild-type pups were subjected to the same NEC-induction protocol as detailed above and randomized after birth to either receive a subcutaneous injection of 40 µg kg^−1^ Y85A recIL-37 variant^26^ or vehicle every 12 h (30 min prior to asphyxia/cold stress). At least three independent experiments were performed for all animal experiments except in experiments on intestinal ILC, in which the data on WT, IL-37tg adults, and IL-37tg NEC are from two independent experiments.

RecIL-37 is commercially available. The Y85A recIL-37 variant as well as IL-37tg mice can be made available under standard material transfer agreement-conditions by contacting the authors CANP or MFN.

### Assessment of weight change

The starting weight of pups ranged between 1.19 and 1.57 g. There was no significant difference in starting weight between WT and IL-37tg pups. At the beginning of each feeding time point, all pups were weighed using a mg-calibrated weighing scale.

### Assessment of hematochezia and diarrhea

The stool passed by the pups was assessed visually for hematochezia (based on stool blood quantity) or diarrhea (by stool consistency) on a scale of 0 (normal) to 3 (severe pathology).

### Assessment of ileus

At the time of death or experimental endpoint at 72 h, the abdomen was opened, the small intestine was excised and assessed for ileus on a scale of 0 (normal) to 3 (severe pathology) by abundance/distribution of luminal bubbles inside the small intestine.

### Histological scoring and IHC

Murine sections from the duodenum, jejunum, and ileum (0.5 cm each) were fixed overnight in 4% paraformaldehyde and then embedded in paraffin. Subsequently, 4 µm sections were H&E-stained and scanned on an Aperio Scanscope (Leica Biosystems, Germany) for histological evaluation of intestinal architecture in Aperio ImageScope v12.4.0.5043 (Leica Biosystems). The histological injury was assessed by an experienced pathologist and scored by two evaluators under the pathologist’s guidance. Scores (Supplementary Fig. 1) ranged from 0 (normal) to 3 (mucosal disintegration) and are based on a previously published murine NEC damage scoring system^83^. Pathologists and evaluators were blinded to the strain and treatment groups.

For IHC in human tissue, 4 μm sections were cut, deparaffinized, and stained on a DAKO Autostainer Plus (Agilent Technologies, USA). In brief, antigens were retrieved in 1× DAKO Target Retrieval Solution at 98 °C for 30 min. Endogenous peroxidase was inhibited using DAKO Real Peroxidase Blocking Solution for 10 min followed by 10 min blocking using DAKO Protein Block. Slides were then incubated with mouse anti-human IL-1R8 (A-4, Santa Cruz Biotechnology, USA, cat# sc-271864) or anti-human IL-37 (37D12, eBioscience, USA, cat# 14-7379-82) or IgG isotype control (ThermoFisher Scientific) diluted 1:50 with DAKO Antibody Diluent for 1 h at RT. Secondary antibody incubation with DAKO EnVision+HRP Polymer Anti-mouse was performed at RT for 30 min and developed using DAKO Liquid DAB + Substrate Chromogen for 10 min. Sections were counterstained with DAKO automation Haematoxylin Staining Reagent for 10 min and coverslipped before scanning on an Aperio Scanscope (Leica Biosystems). IHC scoring was based on intensity and distribution of positive DAB staining from a score of 0 (absent) to 3 (high intensity, widely distributed) and was evaluated by two blinded assessors.

### 16S rDNA sequencing

Total DNA was isolated from all samples using the PowerFecal DNA Isolation Kit (Qiagen, Germany) following the manufacturer’s instructions. After DNA concentration measurement on a NanoDrop (ThermoFisher Scientific), 10 ng of the DNA was used as input for PCR amplification of the V4 region of the 16S rDNA. The F515/R806 primer pair (Supplementary Table 3) for the amplification was fused with Golay indices and adapter sequences as described elsewhere^86^. The PCR was performed on an S1000 Thermal Cycler (BIORAD) in 50 μl reactions using the Platinum PCR SuperMix (ThermoFisher Scientific). Thermal conditions included an initial denaturation step (94 °C for 3 min), followed by 35 amplification cycles (94 °C for 45 s; 58 °C for 60 s; 72 °C for 90 s) and a final elongation step at 72 °C for 10 min. PCR products were purified by size-selection on 2% SizeSelect E-Gels (ThermoFisher Scientific) and quantified on D1000 Tapes using a TapeStation 2200 (Agilent Technologies). The libraries were equimolarly pooled and prepared for Illumina Sequencing using the MiSeq Reagent Kit v3 (Illumina) following the manufacturer’s instructions. Run plan and reagents were adapted according to ref. ^86^. Sequencing was performed on a MiSeq apparatus (Illumina) with 281 cycles.

### Analysis of 16S rDNA sequences

The resulting fastq file was quality checked using FastQC^87^. All regions belonging to sequenced adapters, primers or barcodes, as well as regions with phred score less than 20, were trimmed using Trimmomatic version 0.36^88^ with the following parameters: 2 mismatches allowed in seed sequence, simple clip threshold of 10, and a minimum read length after trimming of 40 nucleotides. QIIME (Quantitative Insights Into Microbial Ecology), version 1.9.1^89^, was deployed to, first, demultiplex the trimmed reads according to the used barcode. This resulted in the summary presented in Supplementary Table 4. QIIME scripts were further used for closed reference OTU (operational taxonomic units) picking with default parameters and the greengenes database gg_13_8_otus/rep_set/97_otus.fasta as taxonomy reference to investigate all present bacterial taxonomies in each of the samples. All taxonomies were summarized, bar plots generated, and the alpha diversities (Shannon index) calculated. Furthermore, alpha diversities were compared to identify statistical differences between groups of different treatments using default parameters, the greatest possible depth of rarefactions (10,000), and non-parametric tests (Monte Carlo permutations to determine the *P*-value).

### Real-time PCR sample processing and RNA data analysis

Murine sections from jejunum and ileum (0.5 cm each) were flushed with ice-cold PBS, snap-frozen in RNAlater (ThermoFisher Scientific), and stored at −80 °C. Human intestinal tissues were directly snap-frozen and stored at −80 °C. For subsequent processing, intestinal tissue was homogenized in RNA lysis buffer (Bioline, Australia) using an Ultra-Turrax homogenizer (IKA, Malaysia). The homogenate was centrifuged for 1 min at 11,000 × *g* and the supernatant was used for RNA isolation. RNA was isolated using the RNA Mini Kit (Bioline), quantified with a NanoDrop (ND-100) spectrophotometer (ThermoFisher Scientific), and assessed to have a 260:280 ratio between 1.7 and 1.8. Total RNA was reverse transcribed using the Tetro cDNA Synthesis kit (Bioline) as per the manufacturer’s instructions. Multiplex real-time PCR was performed in duplicates using TaqMan probes (Applied Biosystems, USA; assay identifiers are listed in Supplementary Table 5 for mouse and Supplementary Table 6 for human studies) on the Fluidigm Biomark HD system (Fluidigm Corporation, USA)^90^ according to the manufacturer’s instructions. PCR failed in 4 human control samples and was excluded from RNA data analysis. Gene expression values were normalized to the most stably expressed housekeeping gene across our samples, hypoxanthine phosphoribosyltransferase 1 (*Hprt1*) for murine tissue and actin beta (*ACTB*) for human tissue. Relative expression was quantified using the ΔΔC_T_ method in Microsoft Excel (Microsoft, USA). The single sample with the highest target gene C_T_ value was set as 1, i.e., baseline, termed “lowest expressed gene” (murine tissue, *Ido1*; human tissue, *TNF*) in the pertinent figures, and used as a constant value in the fold-change formula. Fold-changes of 0 indicates the gene was not detectable. In addition to showing differences in relative mRNA abundance within the same gene between treatment groups, this method of display allows the graphs to provide information on differences in relative mRNA abundance between genes. However, it should be noted that due to limitations of the PCR technology, such a comparison between genes should only be used as a rough guide, and more detailed analysis such as fold-change calculation is discouraged.

### Multiplex ELISA

Segments from the ileum (0.5 cm each) were flushed with ice-cold PBS, snap-frozen in liquid nitrogen, and stored at −80 °C. Ileal tissue was homogenized in lysis buffer^91^ using an Ultra-Turrax homogenizer (IKA). The homogenate was centrifuged for 10 min at 11,000 × *g* and the supernatant was assayed for total protein abundance using the BCA assay (ThermoFisher Scientific). Sample protein concentrations were equalized to 200 µg/ml and cytokine abundance measured by multiplex ELISA using the Quantibody Array (RayBiotech, USA) as per manufacturer’s instructions. Quantibody Array slides were scanned using the Genepix 4000B microarray scanner (Molecular Devices, USA). Normalization of cytokine data to total protein was performed with the following formula: raw cytokine data (in pg/ml)/total protein concentration in the lysate (in mg/ml) in Microsoft Excel (Microsoft). All cytokine changes were normalized to 1 mg total protein.

### Mouse flow cytometry

Lamina propria cells were isolated from the small intestine of 3d old neonatal and male adult (8-12 weeks old) mice using the Lamina Propria Dissociation Kit (Miltenyi Biotec). In brief, excised small intestines were cut open longitudinally and washed in ice-cold Hank’s Balanced Salt Solution (HBSS) to remove stool. After washing, intestines were cut into <0.5 cm pieces and placed into HBSS for further cleaning by brief vortexing before proceeding with the manufacturer’s instructions. Single-cell suspensions of lamina propria cells were pelleted by centrifugation and stained with Zombie Aqua Fixable Viability Dye (Biolegend, catalog number 423102, dilution 1:200) in 1× PBS for 15 min at RT. Following a flow buffer wash, Fc receptors were blocked using anti-CD16/CD32 (eBioscience, cat# 14-0161-82). Cells were then surface stained with CD4-BV786 (BD, cat# 536727, 1:400), CD45.2-FITC (eBioscience, cat# 11-0454-82, 1:200), KLRG1-BV711 (BD, cat# 564014, 1:100), NKp46(CD335)-eFluor660 (eBioscience, cat# 50-3351-82, 1:100), TCRβ-PerCPCy5.5 (eBioscience, cat# 45-5961-80, 1:500) and 1:400 of B220-PE-Cy7 (eBioscience, cat# 25-0452-82), CD11b-PE-Cy7 (eBioscience, cat# 25-0112-82), CD11c-PE-Cy7 (eBioscience, cat# 25-0114-82), CD3e-PE-Cy7 (eBioscience, cat# 25-0031-82), Gr-1-PE-Cy7 (eBioscience, cat# 25-5931-82), and TER-119-PE-Cy7 (eBioscience, cat# 25-5921-82) in ice-cold FACS buffer for 30 min. Cells were then fixed using the Fixation/Permeabilization Concentrate and Diluent (eBioscience) for 30 min at RT. Intracellular staining with GATA3-BV421 (Biolegend, cat# 653814, 1:25), T-bet-PE-CF594 (BD, cat# 562467, 1:25) and RORγt-PE (eBioscience, cat# 12-6988-82, 1:200) was performed in permeabilization buffer (eBioscience) for 30 min at 4 °C. At least 300,000 events were acquired per sample for analysis using the BD LSRFortessa X-20 flow cytometer (BD Bioscience). As depicted in Supplementary Fig. 4, live CD45.2^+^ cells were either analyzed for CD4^+^TCRβ^+^ or CD4^−^TCRβ^+^ T cells or further gated for ILC analysis by excluding lineage-positive cells (selecting for CD4^−^TCRβ^−^) and a lineage-positive dump channel with CD11b, CD11c, TER-119, B220, CD3ε and Gr- 1 (collectively referred to as LIN hereafter). Innate lymphoid cells were assigned to the ILC1, ILC2, and ILC3 groups by expression of the following markers: RORγt^−^NKp46^+^Tbet^+^ ILC1, RORγt^−^NKp46^−^KLRG1^−^^/+^GATA3^+^ ILC2, RORγt^+^NKp46^+^ ILC3, NKp46^−^RORγt^+^Tbet^−^ ILC3, and NKp46^−^RORγt^+^Tbet^+^ ILC3. For data analysis, FlowJo V10 software (Treestar) was used.

### Human study participants

Human tissues from cohort 1 used in mRNA and IHC analyses were provided from the Goethe University Hospital Frankfurt (GUHF), Germany. This study and its protocols were carried out in accordance with the recommendations and approval by the GUHF Multiple Institutional Human Review Board (ref #432/15). Written informed consent was obtained from the families of all participants. Human intestinal samples were obtained from preterm neonates undergoing resection for NEC, at the time of stoma closure or from non-NEC surgical controls. Patient details are shown in Supplementary Table 1. Controls were taken at the time of stoma reanastomosis, i.e., absence of clinical and intestinal illness, and included primary diagnoses such as meconium plug/ileus, spontaneous intestinal perforation, Hirschsprung disease (unaffected tissue used), duodenal atresia, and intestinal pseudo-obstruction.

For human blood from cohort 2 (cohort characteristics are shown in Supplementary Table 2), studies were carried out in accordance with the Declaration of Helsinki, Good Clinical Practice guidelines, and guidelines by the National Health and Medical Research Council of Australia (NHMRC). Ethical approvals were obtained from the Human Research Ethics Committee (HREC) at Monash Health, Clayton, Australia (reference 08100B), and the Royal Women’s Hospital, Parkville, Australia (reference 15/18). Written informed consent was obtained from the families of all participants. Preterm infants were recruited from two tertiary birthing centers with co-located neonatal intensive care units (NICU) within Victoria, Australia. These centers were Monash Newborn/ Monash Medical Centre, Clayton, and the Royal Women’s Hospital, Parkville. Informed consent was obtained from the parents of each infant. Infants were not approached and excluded if they had any major congenital abnormalities or if imminent demise shortly after birth was likely. The cord blood of term infants was collected from infants born between 37 weeks and 0 days (37^+0^) and 41^+0^ weeks of gestation were recruited from Monash Medical Centre following parental consent. Postnatal peripheral blood samples of term infants were collected at Monash Children’s Hospital, Clayton, Victoria, from infants born between 37^+0^ and 41^+0^ weeks that were between 4 and 16 weeks old when undergoing surgery for conditions that are not associated with systemic inflammation (e.g., surgery for hernia repair). To qualify as a healthy term control, infants had to have no health concerns at birth and no maternal or perinatal medical history that could affect their health, including but not limited to intrauterine growth restriction, pre-eclampsia, chorioamnionitis or other maternal infections, gestational diabetes, pre-existing asthma or thyroid disease. The same exclusion criteria applied to the postnatal group. Peripheral blood was collected from healthy adults aged between 20 and 50 years at Hudson Institute of Medical Research, Clayton, who were not on any long-term medications or any anti-inflammatory medication a week prior to sampling.

### Human flow cytometry

500 µl to 3.5 ml of blood samples, depending on sampling time points, were drawn from participants into sodium citrate tubes. For preterm infants, cord blood was collected at birth and peripheral blood collected between 8 and 16 h of life (this sample is known as the day 1 sample), around weeks 1 and 2 and 36 weeks corrected gestational age from an indwelling arterial catheter or a peripheral vein.

Citrated blood samples from infants or adults were centrifuged at 300 × *g* for 15 min at room temperature (RT) onsite in hospital laboratories within 2 h of sample collection, followed by removal of plasma. The remaining blood was diluted 1:4 in culture media [RPMI 1640 (Gibco) with 1% human serum (Sigma-Aldrich, USA) and 1:500 Mycozap Plus-PR (Lonza, Switzerland)] and cultured in 12 ml sterile polypropylene tubes (Greiner Bio-One, Austria) at 37 °C, 5% CO_2_ overnight in the presence of 2 µg/ml brefeldin A (BFA) (Sigma-Aldrich). For antibody staining, cells were washed with PBS once and incubated with eBioscience Human Fc Receptor Binding Inhibitor (Invitrogen, USA) for 20 min at RT. Cells were surface stained with Fixable Viability Dye eFluor 780 (eBioscience, cat# 65-0865-14, 1:2000), CD45-BUV805 (BD, cat# 564914, 1:60), CD66b-BB515 (BD, cat# 564679, 1:40), CD3-BUV496 (BD, cat# 564809, 1:40), CD19-BV786 (BD, cat# 563325, 1:40), CD56-PECy5.5 (eBioscience, cat# 35-0567-42, 1:60), CD14-BUV395 (BD, cat# 563561, 1:30) and CD16-BV510 (BD, cat# 563830, 1:60) for 30 min at RT in the dark. Thereafter, each tube was fixed and permeabilized with eBioscience FoxP3/ transcription factor staining buffer set (Invitrogen) for 30 min at RT in the dark before being washed once with 1× Permeabilization Buffer (Invitrogen). IL-37-PE (eBioscience, cat# 12-7379-42, 1:50) intracellular staining was then incubated for 1 h at RT. Samples were then washed once with flow buffer and stored in the dark at 4 °C until acquisition. As depicted in Supplementary Fig. 8a, IL-37^+^ cells were gated under viable CD45^+^ cells. For cell population analysis, live CD45^+^ cell types were classified in the depicted order (Supplementary Fig. 8b), neutrophils, CD66b^+^; T cells, CD66b^−^CD3^+^; B cells, CD66b^−^CD3^−^CD19^+^; NK cells, CD66b^−^CD3^−^CD19^−^CD56^+^; and monocytes subgroups, CD66b^−^CD3^−^CD19^−^CD56^−^, classical CD14^+^CD16^−^; intermediate CD14^+^CD16^+^; non-classical CD14^−^CD16^+^. At least 50,000 events were acquired per sample for analysis using the BD LSR II (BD Biosciences). Data were analyzed using FlowJo V10 software (TreeStar).

### Statistics

Groups were tested for normality and equal variance (*P* to reject 0.05) using Sigma Plot 14 (Systat Inc., USA). Thereafter, a two-tailed Student’s *t*-test or Mann–Whitney rank-sum test was performed as appropriate, or for multi-group analysis, a one-way ANOVA or one-way ANOVA on ranks was applied to test for significant differences. Where ANOVA revealed significance, post hoc Student–Newman–Keuls or Dunn’s comparisons were performed (the threshold for significance *P* < 0.05). For comparisons between survival curves, a Mantel–Cox log-rank test was performed as appropriate (the threshold for significance *P* < 0.05) using GraphPad Prism 8 (GraphPad Software, USA). A two-tailed Student’s *t*-test or Mann–Whitney rank-sum test was performed as appropriate between dam-fed controls or between adult mice. Where no significant difference was observed (the threshold for significance *P* < 0.05), data were merged for further statistical tests. As adult mice were only assessed in the flow cytometry data sets (Figs. 4, 5b, c and Supplementary Figs. 4, 5h, i), statistical tests as described were run twice, namely first including the adult mice, then also without them to ensure comparability with the other data sets. For human NEC, a two-tailed Student’s *t*-test or Mann–Whitney rank-sum test was performed as appropriate between afflicted and necrotic sections. Where no significant difference was observed (the threshold for significance *P* < 0.05), data were merged for further statistical tests. *r*^2^ values for correlation analyses were calculated by Pearson correlation in Tableau (Tableau Software, USA).

### Reporting summary

Further information on research design is available in the Nature Research Reporting Summary linked to this article.

## Supplementary information

Supplementary Information

Reporting Summary

## Data Availability

The microbiome sequence data have been deposited in the Sequence Read Archive (SRA) at National Center for Biotechnology Information (NCBI) under the accession code SRP133359. All other data are available from the corresponding author upon reasonable request. RecIL-37 is commercially available. The Y85A recIL-37 variant can be made available under standard material transfer agreement-conditions by contacting the authors C.A.N.-P. or M.F.N. Source data are provided with this paper.

## Source data

Source Data
