# Supplementary material for: Characterization of the pathoimmunology of necrotizing enterocolitis reveals novel therapeutic opportunities
Source: Nat Commun. 2020 Nov 13;11:5794. doi: 10.1038/s41467-020-19400-w (PMC7666196; doi:10.1038/s41467-020-19400-w)
Supplement: Supplementary file 1 — Supplementary Information [file 41467_2020_19400_MOESM1_ESM.pdf]

Supplementary Information

accompanying

Characterization of the Pathoimmunology of Necrotizing Enterocolitis Reveals Novel Therapeutic Opportunities

S. X. Cho, C. A. Nold-Petry, M. F. Nold et al.

Supplementary Figures and Legends

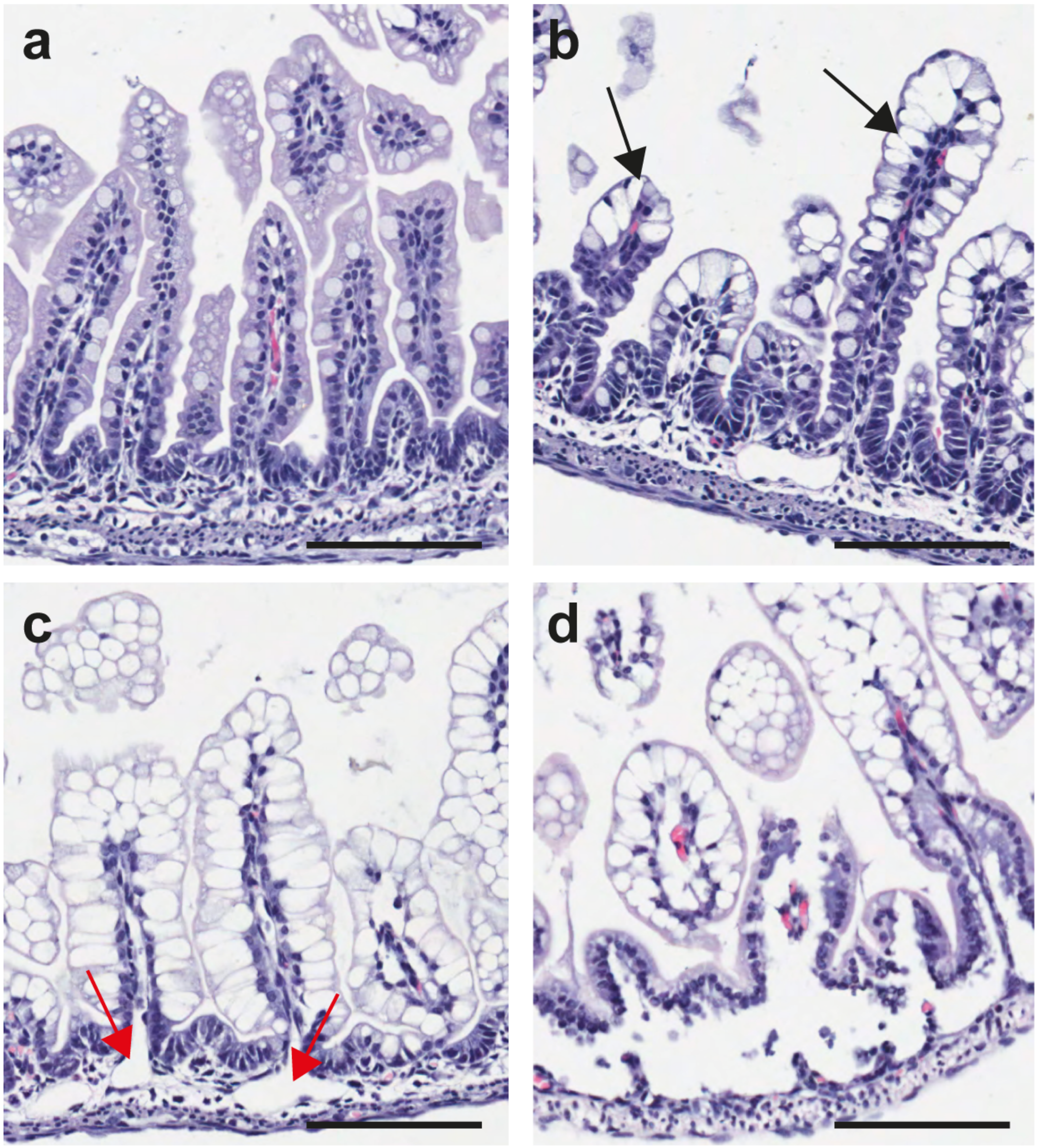

Supplementary Figure 1. Histological scoring of the intestine in neonatal mice. 4µm sections were stained with hematoxylin and eosin showing representative photomicrographs for each morphologic grading score. **a** Score 0, normal. **b** Score 1, moderate enterocyte vacuolation (denoted by black arrows). **c** Score 2, severe enterocyte vacuolation with edema in the lamina propria (denoted by red arrows). **d** Score 3, severe villi enterocyte vacuolation and mucosal disintegration. Magnification 200x; scale bars indicate 50µm.

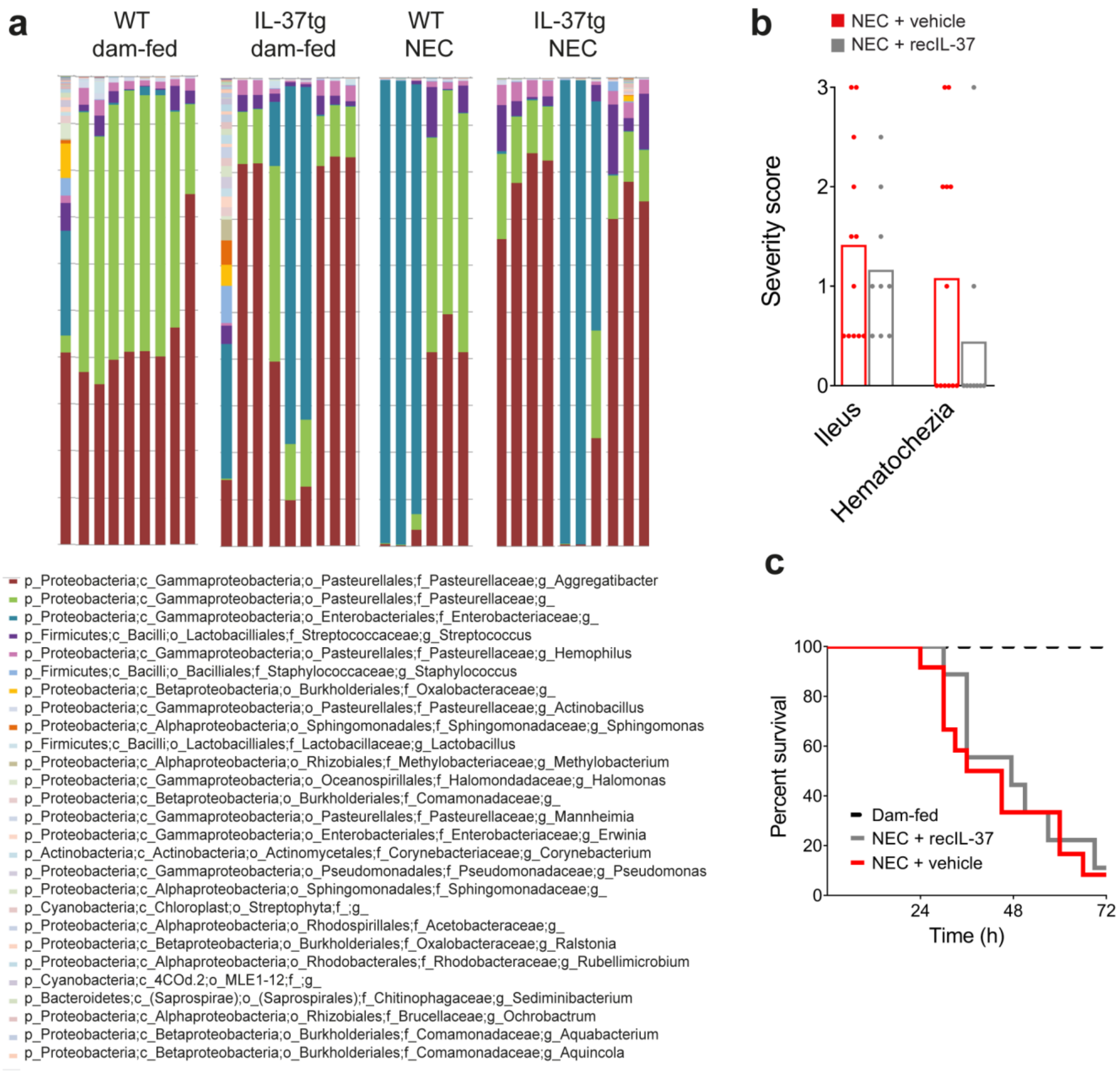

Supplementary Figure 2. Summary of distribution of microbial taxonomies identified in mouse pups and effects of recIL-37 injection on clinical symptoms and survival. a Microbial DNA from cecal tissue samples of some of the newborn WT and IL-37tg mouse pups shown in Figs. 2-5 (n=9 pups for both WT and IL-37tg dam-fed, n=6 for WT NEC and n=10 for IL-37tg NEC) were subjected to the NEC model, followed by sequencing and identification of taxonomy distributions. Data are from 2-4 independent experiments. **b, c** Clinical symptoms **b** and survival **c** were analyzed in the same mice shown in Fig. 2i, i.e. pups injected with 40µg/kg of recIL-37 or vehicle 12-hourly that underwent the NEC model. n=12 pups for NEC+vehicle, 9 for NEC+recIL-37. **b** Mean severity scores of symptoms on a 0-3 scale (no to severe pathology, see Methods) are shown as bars; dots show data points in individual pups. **c** Percent survival is depicted.

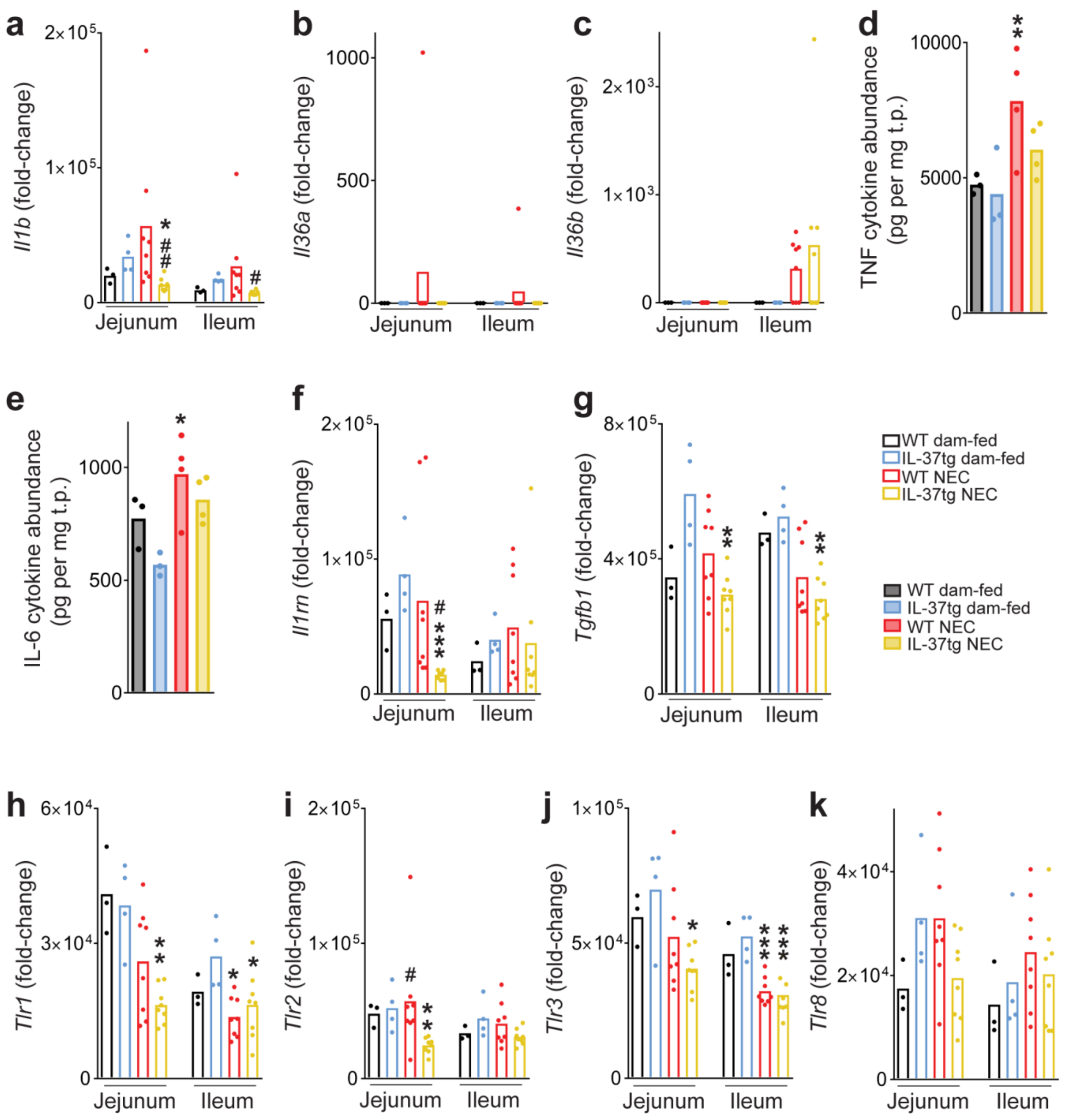

Supplementary Figure 3. Other pro- and anti-inflammatory mediators and TLRs in murine NEC. The same intestinal tissue lysates as in Fig. 3 were measured for gene expression by multiplex real-time PCR (**a-c, f-k**) or protein abundance by ELISA (**d, e**). Dots indicate data from individual mice and bars indicate means. Data are from 2-3 independent experiments; one-way ANOVA or one-way ANOVA on ranks *P* values: *, *P*<0.05; **, *P*<0.01; ***, *P*<0.001 for IL-37tg or WT NEC compared to dam-fed controls (see Methods). #, *P*<0.05; ##, *P*<0.01 for WT NEC compared to IL-37tg NEC. **a-c, f-k** Real-time PCR results of the indicated genes were normalized to *Hprt1* and are shown as fold-change relative to the lowest expressed gene. n=3 pups for WT dam-fed, 4 for IL-37tg dam-fed, 8 for both WT and IL-37tg NEC. **d, e** Ileal cytokine abundance of the indicated protein normalized to total protein (see Methods) is shown. n=3 for both WT and IL-37tg dam-fed, n=4 for both WT and IL-37tg NEC.

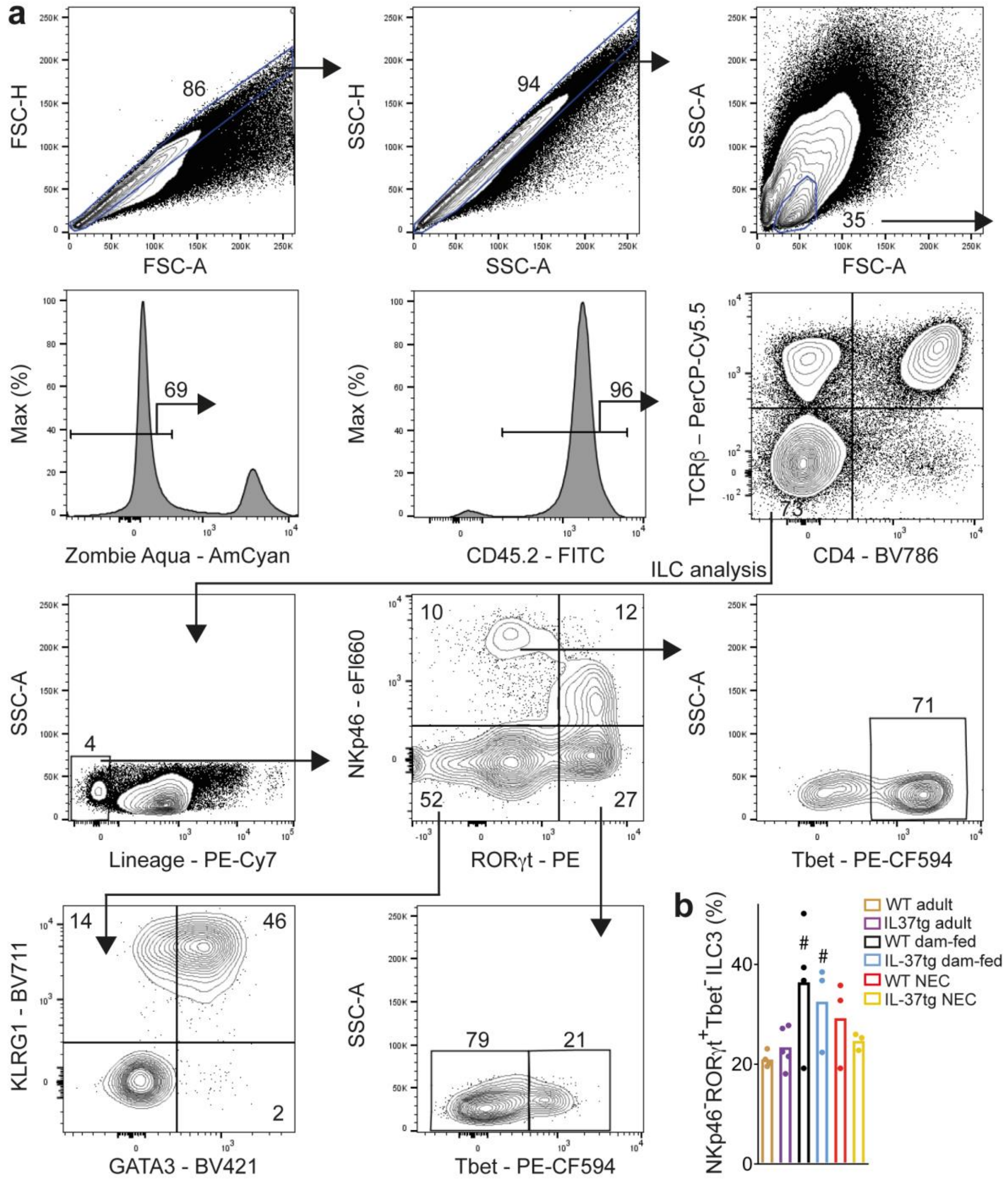

Supplementary Figure 4. Murine intestinal T cells and ILC. Flow cytometric analysis was performed on the same cells as in Fig. 4, Fig. 5b-c and Supplementary 5h-i, i.e. lamina propria cells from the small intestine. Data are from n=2-3 independent experiments. n=4 mice for WT adult, 5 for IL-37tg adult; and n=4 pups for WT dam-fed and 3 each for IL-37tg dam-fed, WT and IL-37tg NEC. (a) Gating strategy for ILC flow analysis. Doublets were excluded, lymphocytes were then gated for further analysis. Live $CD45^+$ cells were then selected for $CD4^-TCR\beta^+$ or $CD4^+TCR\beta^+$ T cells shown in Fig. 5b-c and Supplementary Fig. 5h-i, respectively. For ILC data shown in Fig. 4 and Supplementary Fig. 4b, $TCR\beta^-CD4^-LIN^-$ cells were further analyzed for the expression of NKp46 and RORγt to allow for the discrimination of $ROR\gamma t^-NKp46^+Tbet^+$ ILC1, $ROR\gamma t^-NKp46^-KLRG1^{-/+}GATA3^+$ ILC2, $ROR\gamma t^+NKp46^+$ ILC3, $NKp46^-ROR\gamma t^+Tbet^-$ ILC3 and $NKp46^-ROR\gamma t^+Tbet^+$ ILC3. (b) Percentage of $NKp46^-ROR\gamma t^+Tbet^-$ ILC3 is shown as data points from individual mice (dots) and means (bars). One-way ANOVA *P* values: #, $P<0.05$ for dam-fed compared to adults (see Methods).

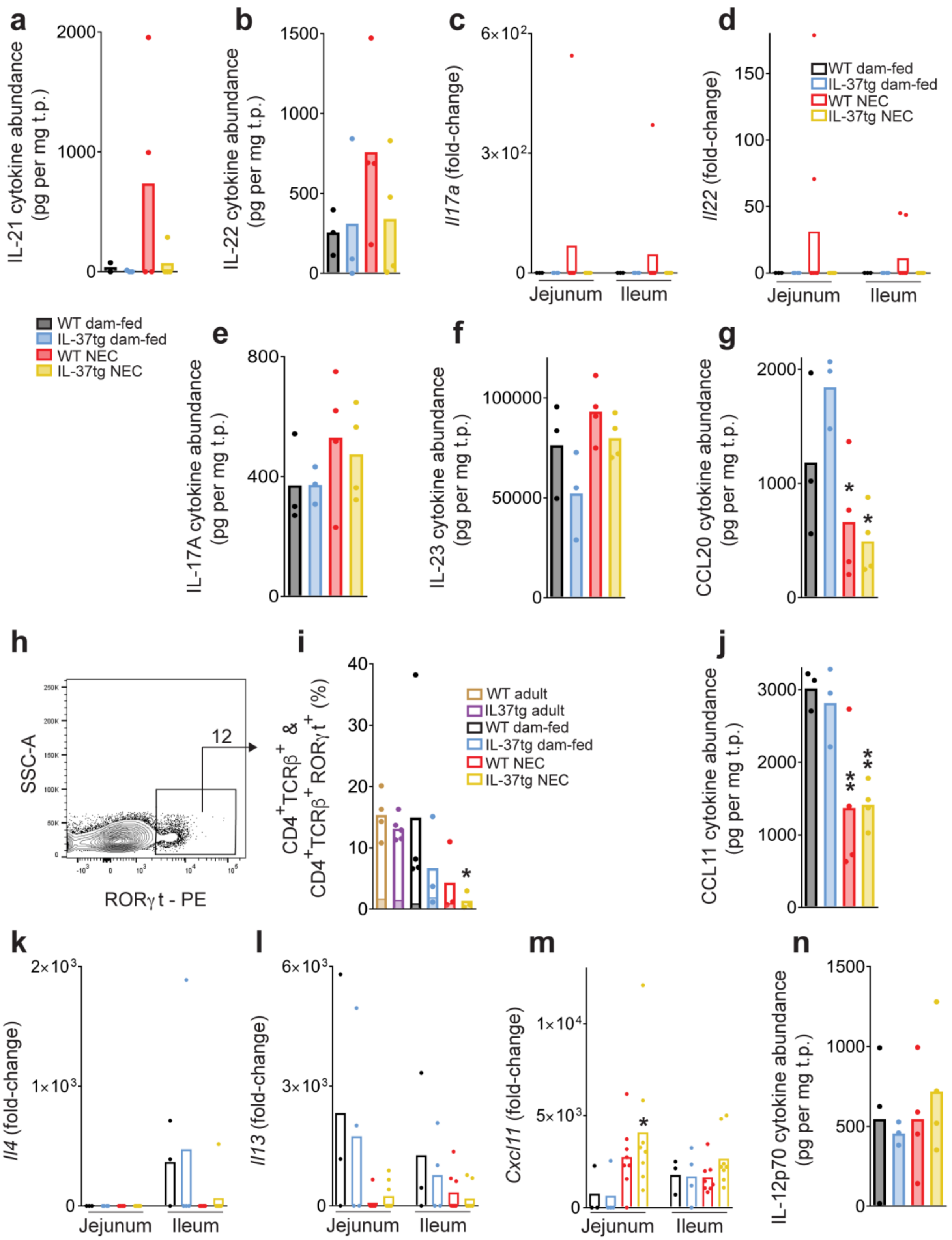

Supplementary Figure 5. Other mediators of adaptive immunity in NEC. The same intestinal tissue lysates as in Figs. 3 and 4 were assayed for gene expression by multiplex real-time PCR (open bars) and protein abundance either by ELISA (filled bars) or flow cytometry. Mediators prototypically belong to the adaptive immunity categories type 3 (**a-i**), type 2 (**j-l**) or type 1 (**m, n**). **a-g, j-n** Data are from 2-3 independent

experiments; for details, see Source Data file. ANOVA on ranks *P* values: *, $P<0.05$ and **, $P<0.01$ for IL-37tg or WT NEC compared to dam-fed controls. **a, b, e-g, j, n** Ileal protein abundance of the indicated mediators is depicted as individual measurements (dots) and means (bars) of cytokine abundance normalized to total protein (t.p.). n=3 pups for both WT and IL-37tg dam-fed, 4 for both WT and IL-37tg NEC. **c, d, k-m** Real-time PCR results for the indicated genes were normalized to *Hprt1* and are depicted as fold-change relative to the lowest expressed gene. Bars show means, dots indicate data points from individual pups. n=3 pups for WT dam-fed, 4 for IL-37tg dam-fed, 8 for both WT and IL-37tg NEC. **h, i** Flow cytometric analysis for T cells on the same cells as in Fig. 4 was performed. **h** Representative gating plot for $CD4^+TCR\beta^+ROR\gamma t^+$ cells, which originate from the live $CD45^+$ lymphocyte gate; arrows indicate source of the accompanying solid color fill in graphs. **i** Graphs show $CD4^+TCR\beta^+$ cells under live $CD45^+$ lymphocytes as mean percentage (bars) and data points from individual mice (dots). One-way ANOVA or ANOVA on ranks *P* value: **, $P<0.01$ for IL-37tg NEC compared to adult (see Methods). n=4 mice for WT adult, 5 for IL-37tg adult; and n=4 pups for WT dam-fed and 3 each for IL-37tg dam-fed, WT and IL-37tg NEC. The solid color fill in represents the mean percentage within each bar that is $ROR\gamma t^+$.

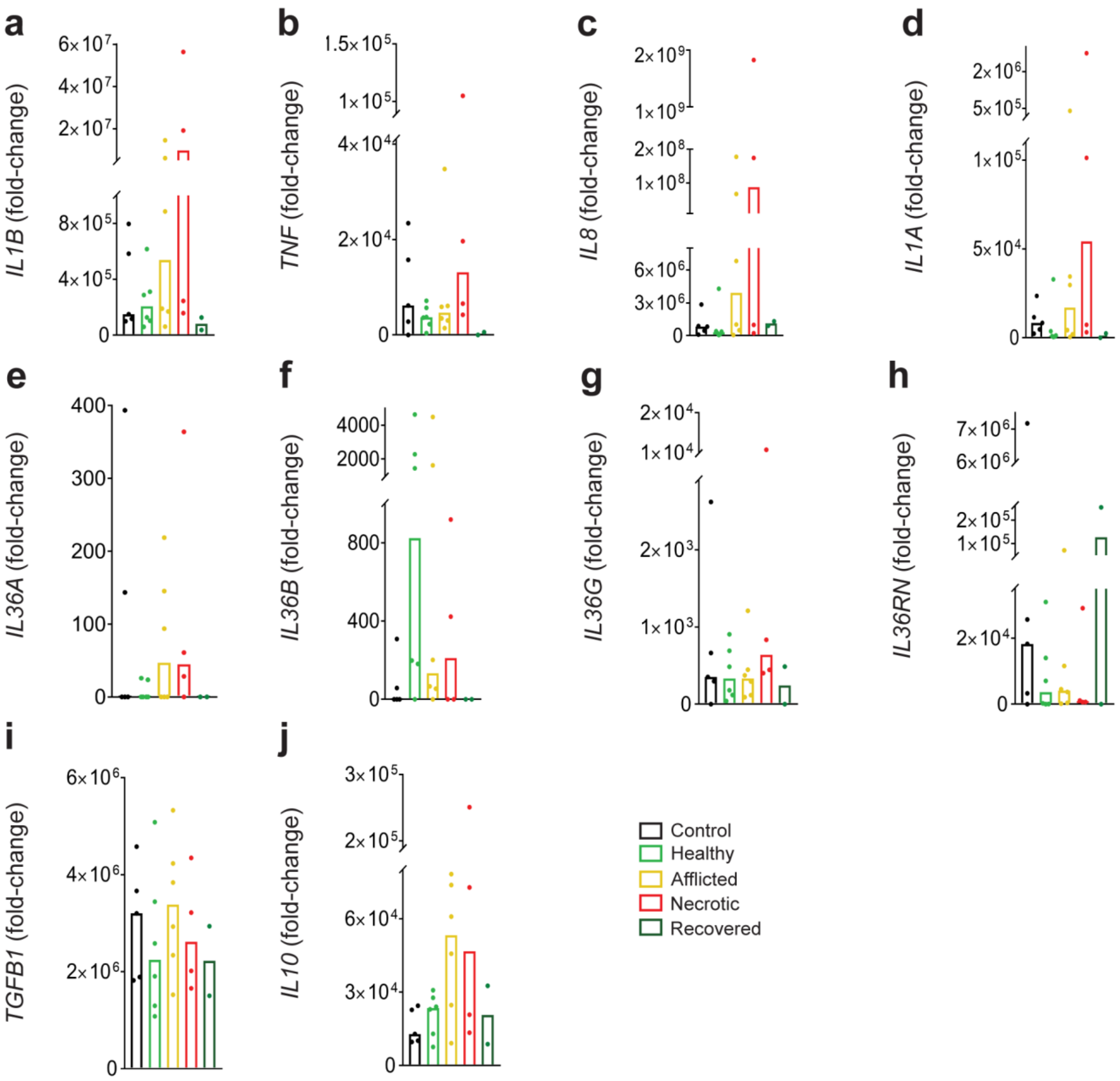

Supplementary Figure 6. Other markers of innate immunity in human NEC. Expression of pro- (**a-g**) and anti- (**h-j**) inflammatory innate immune mediators was determined by multiplex real-time PCR in the same intestinal tissue sections from acute (n=6 healthy/afflicted, n=4 necrotic) and recovered (n=2) NEC as well as non-NEC controls (n=5) as in Fig. 6. Real-time PCR results for the indicated genes were normalized to *ACTB* and depicted as fold-change relative to the lowest expressed gene; dots indicate data from individual patients and bars indicate medians.

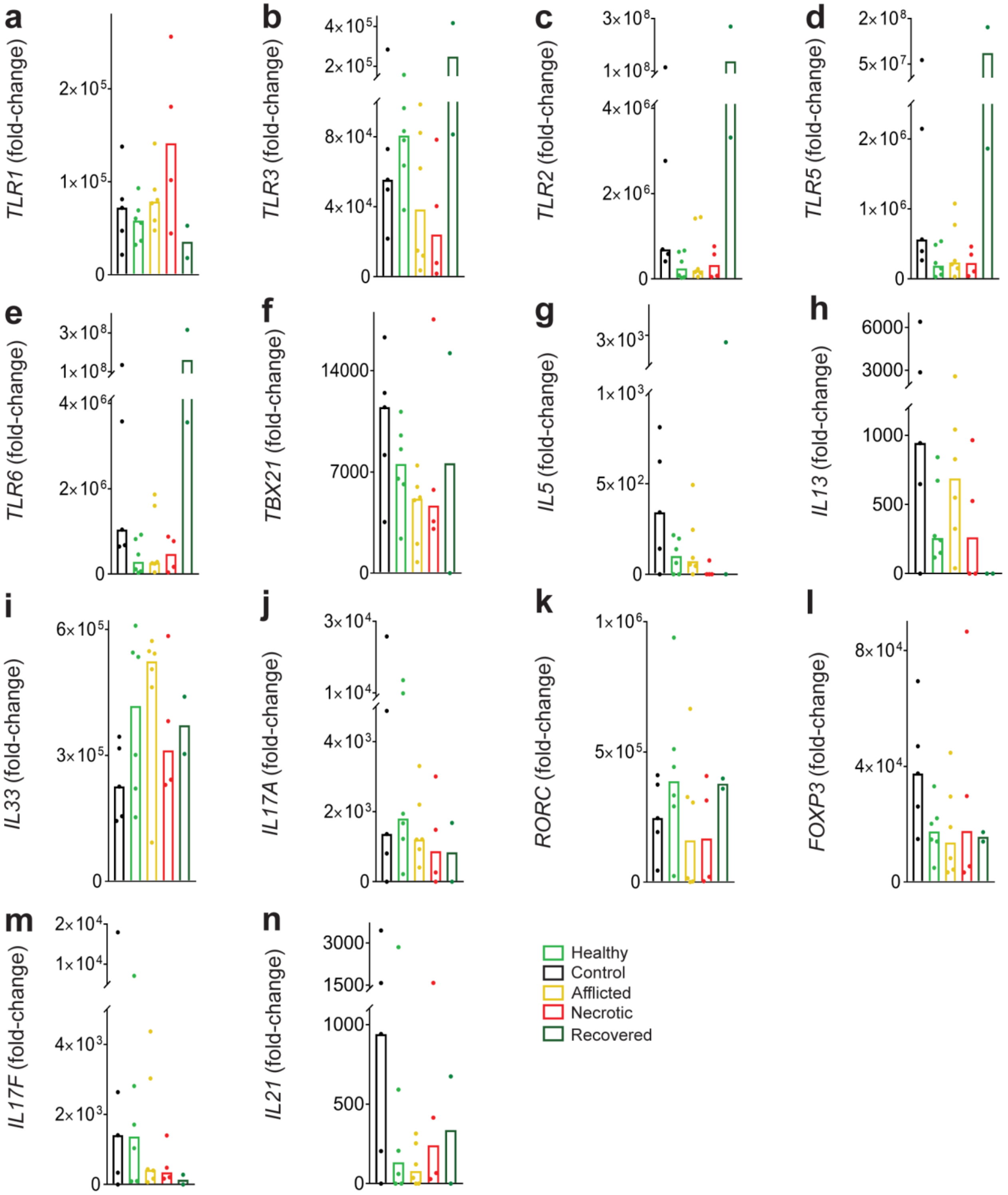

Supplementary Figure 7. Other innate and adaptive Toll-like receptors in human NEC. Expression of Toll-like receptors (**a-e**) as well as adaptive immune type 1 (**f**), type 2 (**g-i**), type 3 (**j, k, m, n**) and Treg (**l**) polarization was determined by multiplex real-time PCR in the same intestinal tissue sections from acute (n=6 healthy/afflicted, n=4 necrotic) and recovered (n=2) NEC as well as non-NEC controls (n=5) as in Fig. 6. Real-time PCR results for the indicated genes were normalized to *ACTB* and depicted as fold-change relative to the lowest expressed gene; data are means (bars) and individual PCR results (dots).

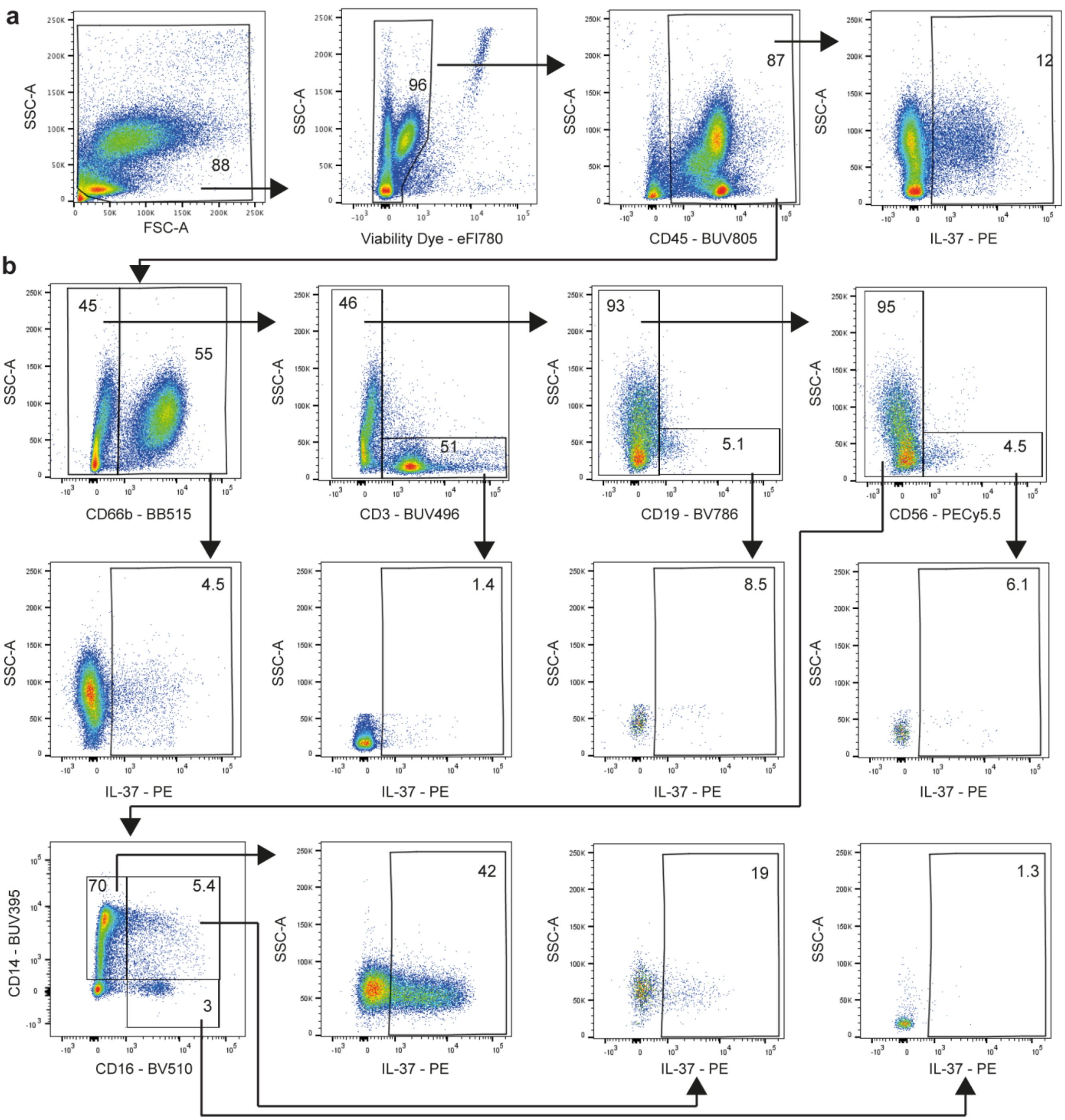

Supplementary Figure 8. Gating strategy for flow cytometry data analysis from cohort 2. Peripheral blood was obtained from a second cohort of premature infants (gestational age 24-29 weeks, n=21), subjected to flow cytometry and analyzed to produce the data shown in Fig. 8 using the gating strategies presented here. **a** Doublets and debris were excluded. Live (identified by fixable viability dye) CD45$^+$ cells were then gated for IL-37$^+$ cells as shown (pertinent data in Fig. 8a-b). **b** For further investigation of IL-37 within subpopulations, cells were gated under live CD45$^+$ cells in the order depicted in **a**, then further subgated for neutrophils (CD66b$^+$), T cells (CD66b$^-$CD3$^+$), B cells (CD66b$^-$CD3$^-$CD19$^+$), NK cells (CD66b$^-$CD3$^-$CD19$^-$CD56$^+$) or monocytes (pertinent data presented in Fig. 8c-h). Percentages are indicated by numbers within or next to boxed fields.

Supplementary Tables and Table Legends

	Gestational age at birth (weeks +days)	Weight at birth (g)	Age at operation (days)	Weight at operation (g)	Localization of ileostoma proximal to ileocecal junction (cm)
Control 1	33+6	2240	78	2340	10
Control 2	25+2	740	102	2600	1
Control 3	35+2	1800	252	5200	10
Control 4	25+4	630	150	3740	15
Control 5	24+3	740	119	2500	10
Control 6	25+5	860	59	1100	10
Control 7	28+1	640	76	2000	10
Control 8	39+4	4190	31	4300	30
Control 9	25+6	845	104	2300	2
NEC 1	26+5	950	24	1290	20
NEC 2	25+5	735	49	1400	8
NEC 3	25+0	550	10	585	No stoma
NEC 4	23+4	470	27	670	5
NEC 5	26+1	680	38	1140	No stoma
NEC 6	24+4	740	12	815	7
Recovered 1 (NEC 2)	25+5	735	111	2200	10
Recovered 2 (unmatched)	27+2	1047	155	4780	8

Supplementary Table 1. Human patient details cohort 1. All NEC infants were Stage III. Controls 1, 3, 5, 8 were excluded from RNA analysis due to failure of RNA isolation/PCR.

	Absolute value	% or SD/IQR	No NEC	% or SD/IQR	NEC	% or SD/IQR	*P* non-NEC vs NEC
Study participants	21	n/a	16	76%	5	24%	n/a
Prenatal steroid use (%)	21	100%	16	100%	5	100%	1
Prenatal steroid use (median doses)	2		2		2		0.926
PPROM (%)	9	43%	7	44%	2	40%	0.923
Cesarean section (%)	15	71%	10	63%	5	100%	0.126
Histopathological evidence of chorioamnionitis (%)	11	52%	9	56%	2	40%	0.567
IUGR (%)	5	24%	2	13%	3	60%	0.039
Birth weight (mean ± SD, g)	937	176	965	189	850	89	0.342
GA (mean ± SD, weeks+days)	27+0	9.5	26+6	9.6	27+1	10.1	0.772
Male (%)	12	57%	8	50%	4	80%	0.268
1 min Apgar score (median-IQR)	5	(3-6.5)	5	(3-6)	5	(2-7)	0.809
5 min Apgar score (median-IQR)	8	(5.5-8.5)	7	(5.3-8)	8	(4-9.5)	0.586
Severe ICH/IVH (Grade III-IV, %)	1	5%	1	6%	0	0%	0.655
Early-onset sepsis (First 72 h, %)	6	29%	5	31%	1	20%	0.673
Late-onset sepsis (After 72h, %)	9	43%	5	31%	4	80%	0.068

Supplementary Table 2. Human patient details cohort 2. Of the 5 NEC infants, there were 3x Stage I, 1x Stage II and 1x Stage III. NEC, necrotizing enterocolitis; PPROM, preterm premature rupture of the membranes; IUGR, intrauterine growth restriction; GA, gestational age; ICH/IVH, intracranial/intraventricular hemorrhage.

**Forward construct Primer name/region**	**Sequence**
Full Primer Sequence (Fw-V4)	AATGATACGGCGACCACCGAGATCTACACTATGGTAATTGTGTGC CAGCMGCCGCGGTAA
5’ illumina adapter	AATGATACGGCGACCACCGAG
Forward pad	ATCTACACTATGGTAATT
Forward linker	GT
Fw primer sequence (515F)	GTGCCAGCMGCCGCGGTAA
**Reverse construct Primer name/region**	**Sequence**
Full Primer Sequence (Rv-V4)	CAAGCAGAAGACGGCATACGAGAT(12mer_Golay_Barcode)AGTC AGTCAGCCGGACTACHVGGGTWTCTAAT
3’ illumina adapter	CAAGCAGAAGACGGCATACGAGAT
Reverse pad	AGTCAGTCAG
Reverse linker	CC
Rv primer sequence (806R)	GGACTACHVGGGTWTCTAAT
**Sequencing Primer name**	**Sequence**
Read 1 primer	TATGGTAATTGTGTGYCAGCMGCCGCGGTAA
Read 2 primer	AGTCAGCCAGCCGGACTACNVGGGTWTCTAAT
Index primer	AATGATACGGCGACCACCGAGATCTACACGCT

Supplementary Table 3. Forward and reverse primer constructs for 16S rDNA-V4 PCR.

Sample ID	Number of assigned reads
WT DF 1	58,037
WT DF 2	108,067
WT DF 3	114,198
WT DF 4	71,795
WT DF 5	76,406
WT DF 6	113,711
WT DF 7	101,554
WT DF 8	132,941
WT DF 9	116,047
IL-37tg DF 1	89,570
IL-37tg DF 2	65,967
IL-37tg DF 3	97,403
IL-37tg DF 4	147,021
IL-37tg DF 5	63,591
IL-37tg DF 6	59,960
IL-37tg DF 7	106,815
IL-37tg DF 8	95,747
IL-37tg DF 9	153,964
WT NEC 1	78,736
WT NEC 2	81,347
WT NEC 3	119,952
WT NEC 4	71,672
WT NEC 5	84,780
WT NEC 6	90,556
IL-37tg NEC 1	81,174
IL-37tg NEC 2	56,958
IL-37tg NEC 3	41,811
IL-37tg NEC 4	116,589
IL-37tg NEC 5	114,931
IL-37tg NEC 6	103,084
IL-37tg NEC 7	116,618
IL-37tg NEC 8	112,110
IL-37tg NEC 9	142,160
IL-37tg NEC 10	69,929
Total number seqs	3,255,201

Supplementary Table 4. Overview of reads assigned to each of the samples after multiplexing.

Mouse assays	
**Gene name**	**Assay ID**
B2m	Mm00437762_m1
Cxcl1	Mm04207460_m1
Cxcl10	Mm00445235_m1
Cxcl11	Mm00444662_m1
Cxcr3	Mm00438259_m1
Foxp3	Mm00475162_m1
Gapdh	Mm99999915_g1
Gata3	Mm00484683_m1
Hprt1	Mm00446968_m1
Ido1	Mm00492586_m1
Il1b	Mm00434228_m1
Il4	Mm00445259_m1
Il6	Mm00446190_m1
Il13	Mm00434204_m1
Il17a	Mm00439618_m1
Il22	Mm00444241_m1
Il33	Mm00505403_m1
Il1f6 (Il36a)	Mm00457645_m1
Il1f8 (Il36b)	Mm01337546_g1
Il1f9 (Il36g)	Mm00463327_m1
Il1rn	Mm00446186_m1
Tbx21 (Tbet)	Mm00450960_m1
Tgfb1	Mm00441724_m1
Tlr1	Mm00446095_m1
Tlr2	Mm00442346_m1
Tlr3	Mm01207404_m1
Tlr4	Mm00445273_m1
Tlr5	Mm00546288_s1
Tlr6	Mm02529782_s1
Tlr7	Mm00446590_m1
Tlr8	Mm04209873_m1
Tlr9	Mm00446193_m1
Tlr11	Mm01701924_s1
Tlr12	Mm01180204_s1
Tlr13	Mm01233819_m1

Supplementary Table 5. Taqman gene expression assays mouse studies.

Human assays	
**Gene name**	**Assay ID**
ACTB	Hs01060665_g1
CXCL10	Hs00171042_m1
CXCL11	Hs00171138_m1
CXCR3	Hs01847760_s1
FOXP3	Hs01085834_m1
GAPDH	Hs02786624_g1
GATA3	Hs00231122_m1
HPRT1	Hs02800695_m1
IDO1	Hs00984148_m1
IFNG	Hs00989291_m1
IL1A	Hs00174092_m1
IL1B	Hs01555410_m1
IL5	Hs01548712_g1
IL6	Hs00174131_m1
IL8	Hs00174103_m1
IL10	Hs00961622_m1
IL13	Hs00174379_m1
IL17A	Hs00174383_m1
IL17F	Hs00369400_m1
IL17RA	Hs01056316_m1
IL21	Hs00222327_m1
IL22	Hs01574154_m1
IL33	Hs00369211_m1
IL1F5 (IL36RN)	Hs01104220_m1
IL1F6 (IL36A)	Hs00205367_m1
IL1F8 (IL36B)	Hs00758166_m1
IL1F9 (IL36G)	Hs00219742_m1
NLRP3	Hs00918082_m1
RORC	Hs01076112_m1
TBX21 (TBET)	Hs00894392_m1
TGFB1	Hs00998133_m1
TLR1	Hs00413978_m1
TLR2	Hs02621280_s1
TLR3	Hs01551079_g1
TLR4	Hs00152939_m1
TLR5	Hs01920773_s1
TLR6	Hs01039989_s1
TLR7	Hs01933259_s1
TLR8	Hs00152972_m1
TLR9	Hs00370913_s1
TLR10	Hs01935337_s1
TNF	Hs00174128_m1

Supplementary Table 6. Taqman gene expression assays human studies.
