# Supplementary material for: Characterization of the pathoimmunology of necrotizing enterocolitis reveals novel therapeutic opportunities
Source: Nat Commun. 2020 Nov 13;11:5794. doi: 10.1038/s41467-020-19400-w (PMC7666196; doi:10.1038/s41467-020-19400-w)
Supplement: Supplementary file 2 — Reporting Summary [file 41467_2020_19400_MOESM2_ESM.pdf]

nature research

Corresponding author(s): Marcel F Nold

Last updated by author(s): Sep 27, 2020

Reporting Summary

Nature Research wishes to improve the reproducibility of the work that we publish. This form provides structure for consistency and transparency in reporting. For further information on Nature Research policies, see Authors & Referees and the Editorial Policy Checklist .

Statistics

For all statistical analyses, confirm that the following items are present in the figure legend, table legend, main text, or Methods section.

n/a	Confirmed	
☐	☒	The exact sample size (*n*) for each experimental group/condition, given as a discrete number and unit of measurement
☐	☒	A statement on whether measurements were taken from distinct samples or whether the same sample was measured repeatedly
☐	☒	The statistical test(s) used AND whether they are one- or two-sided<br>*Only common tests should be described solely by name; describe more complex techniques in the Methods section.*
☒	☐	A description of all covariates tested
☐	☒	A description of any assumptions or corrections, such as tests of normality and adjustment for multiple comparisons
☐	☒	A full description of the statistical parameters including central tendency (e.g. means) or other basic estimates (e.g. regression coefficient) AND variation (e.g. standard deviation) or associated estimates of uncertainty (e.g. confidence intervals)
☐	☒	For null hypothesis testing, the test statistic (e.g. *F*, *t*, *r*) with confidence intervals, effect sizes, degrees of freedom and *P* value noted<br>*Give P values as exact values whenever suitable.*
☐	☒	For Bayesian analysis, information on the choice of priors and Markov chain Monte Carlo settings
☒	☐	For hierarchical and complex designs, identification of the appropriate level for tests and full reporting of outcomes
☐	☒	Estimates of effect sizes (e.g. Cohen's *d*, Pearson's *r*), indicating how they were calculated

Our web collection on statistics for biologists contains articles on many of the points above.

Software and code

Policy information about availability of computer code

Data collection	No custom code or software was used for data collection.
Data analysis	All software used for data analysis are commercially available. FlowJo V10 (Treestar) - flow cytometry data analysis; Microsoft Excel (version 2007) - multiplex ELISA and qPCR data analysis; FastQC (version 0.11.5) and Trimmomatic (version 0.36) - quality control of raw microbial sequence data; QIIME (version 1.9.1) - microbiome data analysis; Graphpad Prism 6 - graph plotting and statistics; Aperio ImageScope 12.4.0.5043 - viewing of histology and immunostained slides; SigmaPlot V14 - statistical testing; and Tableau 2019.1 - Pearson correlations.

For manuscripts utilizing custom algorithms or software that are central to the research but not yet described in published literature, software must be made available to editors/reviewers. We strongly encourage code deposition in a community repository (e.g. GitHub). See the Nature Research guidelines for submitting code & software for further information.

Data

Policy information about availability of data

All manuscripts must include a data availability statement. This statement should provide the following information, where applicable:

- Accession codes, unique identifiers, or web links for publicly available datasets
- A list of figures that have associated raw data
- A description of any restrictions on data availability

The source data underlying Figs 2a-b, e-f, h-i, 3, 4b-c, e, g-h, 5a, c-m, 6b-p, 7a-b, d-e, g-h, 8b, d-e and Supplementary Figs 2b-c, 3, 4b, 5, 6 and 7 are provided in a Source Data file. 16S rRNA fastq sequence files from microbiome analysis are available as stated in Methods under accession number SRP133359 in the Sequence Read Archive at the National Center for Biotechnology (NCBI).

Field-specific reporting

Please select the one below that is the best fit for your research. If you are not sure, read the appropriate sections before making your selection.

☒ Life sciences ☐ Behavioural & social sciences ☐ Ecological, evolutionary & environmental sciences

For a reference copy of the document with all sections, see nature.com/documents/nr-reporting-summary-flat.pdf

Life sciences study design

All studies must disclose on these points even when the disclosure is negative.

Sample size	At the outset of this study, there was limited information available on most mediators we set out to investigate; therefore, sample size estimates to ensure adequate power to detect a pre-determined effect size would have been rather speculative, and we did not use them. Hence, we used PMID 26690704 for orientation of mouse experiment sample sizes, PMIDs 26274503 and 31582728 for human cohort 1, and PMID 26704574 for human cohort 2.
Data exclusions	Four human control samples from cohort 1 were excluded from the RNA analyses due to PCR failure as stated in the Methods. Two human non-NEC preterm infant samples were excluded from the flow cytometry analysis of cohort 2 due to poor cell quality.
Replication	PCR experiments were conducted in technical duplicates, i.e. every sample was measured twice. Average Ct values were calculated from the results of these duplicates to derive the individual data points in the relevant figures. For multiplex protein slide arrays, technical quadruplicates were averaged and depicted in the same fashion. IHC data were generated by obtaining representative images for each patient; these images were scored independently by two blinded assessors. These averages constitute the individual data points in the relevant figures.
Randomization	Mouse data: Animals were randomized into experimental groups.<br>Human data: Randomization was not applicable to cohort 1 because we specifically enrolled infants based on condition. Babies from cohort 2 were consented antenatally and as such NEC status was not known at the time of consent. Covariates considered in cohort 2 are depicted in Supplementary Table 2. P-values were <0.1 only for IUGR and LOS. Given the known strong association between NEC and IUGR as well as LOS, a difference between NEC and non-NEC infants with regard to IUGR and LOS was expected (significant for IUGR, close to significance for LOS, P=0.039 and 0.068, respectively). Therefore, our sample size did not allow us to adequately control for these two covariates; much larger cohort studies will be required to do so.
Blinding	Investigators were blinded to group allocation for mouse experiments during data collection and IHC analysis. Similarly, for human data analysis, investigators were blinded to condition (i.e. NEC vs controls) for both flow cytometry analysis and IHC analysis.

Reporting for specific materials, systems and methods

We require information from authors about some types of materials, experimental systems and methods used in many studies. Here, indicate whether each material, system or method listed is relevant to your study. If you are not sure if a list item applies to your research, read the appropriate section before selecting a response.

Materials & experimental systems

n/a	Involved in the study	
☐	☒	Antibodies
☒	☐	Eukaryotic cell lines
☒	☐	Palaeontology
☐	☒	Animals and other organisms
☐	☒	Human research participants
☒	☐	Clinical data

Methods

n/a	Involved in the study	
☒	☐	ChIP-seq
☐	☒	Flow cytometry
☒	☐	MRI-based neuroimaging

Antibodies

Antibodies used	Flow cytometry (mouse panel):<br>Fc block (CD16/CD32), Company: eBioscience, Clone: 93, Cat#: 14-0161-82<br>Zombie Aqua Fixable Viability Dye, Company: Biolegend, Clone: NA, Cat#423102<br>CD4-BV786, Company: BD, Clone: RM4.5, Cat#: 563727 (company website re validation: "Reactivity: Mouse (QC Testing); Application: Flow cytometry (Routinely Tested)"<br>CD45.2-FITC, Company: eBioscience, Clone: 104, Cat#: 11-0454-82 (website: "Applications Tested: The 104 antibody has been tested by flow cytometric analysis of mouse splenocytes. This can be used at less than or equal to 0.5 ug per test.")<br>GATA3-BV421, Company: Biolegend, Clone: 16E10A23, Cat#: 653814 (website: "Reactivity: Human, Mouse; Recommended Usage: Each lot of this antibody is quality control tested by intracellular immunofluorescent staining with flow cytometric analysis. For flow cytometric staining, the suggested use of this reagent is 5 ul per million cells in 100 ul staining volume [...].")

KLRG1-BV711, Company: BD, Clone: 2F1, Cat#: 564014 (website: "Reactivity: Mouse (QC Testing); Application: Flow cytometry (Routinely Tested)"

B220-PE-Cy7, Company: eBioscience, Clone: RA3-6B2, Cat#: 25-0452-82 (website: "Species Reactivity: Human, Mouse. This antibody was verified by Relative expression to ensure that the antibody binds to the antigen stated." For details on validation by "Relative expression", see https://www.thermofisher.com/au/en/home/life-science/antibodies/invitrogen-antibody-validation/relative-expression-antibody-validation.html)

CD11b-PE-Cy7, Company: eBioscience, Clone: M1/70, Cat#: 25-0112-82 (website: "Applications Tested: The M1/70 antibody has been tested by flow cytometric analysis of mouse splenocytes and bone marrow cells. This can be used at less than or equal to 0.125 ug per test.")

CD11c-PE-Cy7, Company: eBioscience, Clone: N418, Cat#: 25-0114-82 (website: "Applications Tested: This N418 antibody has been tested by flow cytometric analysis of mouse splenocytes. This can be used at less than or equal to 0.5 ug per test.")

CD3e-PE-Cy7, Company: eBioscience, Clone: 145-2C11, Cat#: 25-0031-82 (website: "Applications Tested: This 145-2C11 antibody has been tested by flow cytometric analysis of mouse thymocytes and splenocytes. This can be used at less than or equal to 1 ug per test.")

Gr1-PE-Cy7, Company: eBioscience, Clone: RB6-8C5, Cat#: 25-5931-82 (website: "Species Reactivity: Mouse. This antibody was verified by Relative expression to ensure that the antibody binds to the antigen stated.")

TER119-PE-Cy7, Company: eBioscience, Clone: TER-119, Cat#: 25-5921-82 (website: "Applications Tested: This TER-119 antibody has been tested by flow cytometric analysis of mouse splenocytes and bone marrow cells. This can be used at less than or equal to 0.5 ug per test.")

NKp46(CD335)-eFl660, Company: eBioscience, Clone: 29A1.4, Cat#: 50-3351-82 (website: "Applications Tested: This 29A1.4 antibody has been tested by flow cytometric analysis of mouse splenocytes. This can be used at less than or equal to 1 ug per test.")

RORgammat-PE, Company: eBioscience, Clone: AFKJS-9, Cat#: 12-6988-82 (website: "Applications Tested: This AFKJS-9 antibody has been tested by intracellular staining followed by flow cytometric analysis of mouse thymocytes and Th17-polarized splenocytes using the FoxP3/Transcription Factor Staining Buffer Set and protocol [which we used]. [...] This can be used at less than or equal to 0.5 ug per test.")

T-bet-PE-CF594, Company: BD, Clone: O4-46, Cat#: 562467 (website: "Reactivity: [...] Mouse (Tested in Development); Application: Intracellular staining (Flow cytometry) (Routinely Tested)"

TCRbeta-PerCPCy5.5, Company: eBioscience, Clone: H57-597, Cat#: 45-5961-80 (website: "Applications Tested: This H57-597 antibody has been tested by flow cytometric analysis of mouse splenocytes. This can be used at less than or equal to 0.25 ug per test.")

Flow cytometry (human panel):

Fc Receptor Binding Inhibitor, Company: eBioscience, Clone: Poly, Cat#14-9161-73

Fixable Viability Dye-eFluor 780, Company: eBioscience, Clone: NA, Cat# 65-0865-14

CD45-BUV805, Company: BD, Clone: HI30, Cat#: 564914 (website: "Reactivity: Human (QC Testing); Application: Flow cytometry (Routinely Tested)"

IL37-PE, Company: eBioscience, Clone: 37D12, Cat#: 12-7379-42 (We have developed this clone and antibody together with eBioscience, and in the process of this development have performed extensive validation under a wide range of conditions, including flow cytometry and immunostaining/-fluorescence.)

CD66b-BB515, Company: BD, Clone: G10F5, Cat#: 564679 (website: "Reactivity: Human (QC Testing); Application: Flow cytometry (Routinely Tested)"

CD3-BUV496, Company: BD, Clone: UCHT1, Cat#: 564809 (website: "Reactivity: Human (QC Testing); Application: Flow cytometry (Routinely Tested)"

CD19-BV786, Company: BD, Clone: SJ25C1, Cat#: 563325 (website: "Reactivity: Human (QC Testing); Application: Flow cytometry (Routinely Tested)"

CD56-PE-Cy5.5, Company: eBioscience, Clone: CMSSB, Cat#: 35-0567-42 (website: This CMSSB antibody has been pre-titrated and tested by flow cytometric analysis of normal human peripheral blood cells. This can be used at 5 µL (0.125 µg) per test.)

CD14-BUV395, Company: BD, Clone: MϕP9, Cat#: 563561 (website: "Reactivity: Human (QC Testing); Application: Flow cytometry (Routinely Tested)"

CD16-BV510, Company: BD, Clone: 3G8, Cat#: 563830 (website: "Reactivity: Human (QC Testing) [...]; Application: Flow cytometry (Routinely Tested)"

IHC (anti-human):

IL-1R8, Company: Santa Cruz Biotechnology, Clone: A-4, Cat#: sc-271864 (website: Specific for an epitope mapping between amino acids 377-394 at the C-terminus of SIGIRR of human origin. Anti-SIGIRR Antibody (A-4) is recommended for detection of SIGIRR of mouse, rat and human origin by WB, IP, IF and ELISA.)

IL-37, Company: eBioscience, Clone: 37D12, Cat#: 14-7379-82 (Also see comment on anti-IL-37 antibody above. Website: This 37D12 antibody has been tested by immunocytochemistry of fixed and permeabilized normal human peripheral blood mononuclear cells and can be used at less than or equal to 20 µg/mL.)

DAKO EnVision+HRP polymer anti-mouse secondary antibody, Company: DAKO, Clone: NA, Cat#: K4001

Validation

Flow cytometry: All antibodies are validated by manufacturer for use in flow cytometry and in their specified species as listed in the "Antibodies used" section above. Before use to generate data for this paper, we carefully titrated each of these antibodies individually, and then performed optimization of the full panels.

Immunohistochemistry: Both IL-1R8 and IL-37 antibodies were validated by the manufacturer for use in immunostaining, and were used in several publications, including our own [Nold-Petry, CA., Nat Immunol, 2015]. Specificity of secondary antibodies was validated by use of control slides that did not have primary antibody.

Animals and other organisms

Policy information about studies involving animals; ARRIVE guidelines recommended for reporting animal research

Laboratory animals	C57BL/6J wild-type (WT) mice were originally purchased from Jackson Laboratories (USA) and IL-37 transgenic (IL-37tg) mice were homozygous offspring from the original colonies as published (Nold MF, Nat Immunol, 2010). Both strains were co-housed in the same room in our animal facility. Only mice between 8 weeks and 12 weeks of age were used as breeders. Day 0 - day 3 male and female newborn pups were used in this study. Male 8-12 week old mice of both strains were used as adult controls for mouse flow cytometry experiments.
Wild animals	Study did not involve wild animals.
Field-collected samples	Study did not involve samples collected from the field.
Ethics oversight	All animal work was approved by Monash University's Animal Ethics Committee (MMCA/2012/62, MMCA/2017/30) and was conducted in accordance with the principles of the Declaration of Helsinki.

Note that full information on the approval of the study protocol must also be provided in the manuscript.

Human research participants

Policy information about studies involving human research participants

Population characteristics	See Supplementary Tables 1 and 2 in the manuscript.
Recruitment	For details on recruitment, see Methods under Human study participants. Briefly, the legal guardians of the infants recruited into cohort 1 were approached after the need for surgery was decided upon by the treating clinicians. This was the case for the NEC group as well as the control group. For cohort 2, recruitment occurred prospectively, namely antenatally or in the first hours after birth. Therefore, we deem the influence of any recruitment-related bias on our data minimal, if at all present.
Ethics oversight	Cohort 1: University Hospital Frankfurt (UHF) Multiple Institutional Human Review Board, Germany (ref #432/15)<br>Cohort 2: Human Research Ethics Committee (HREC) at Monash Health, Clayton, Australia (reference: 08100B), Royal Women's Hospital, Parkville, Australia (reference: 15/18), and Mercy Hospital, Heidelberg, Australia (ref R12/66).

Note that full information on the approval of the study protocol must also be provided in the manuscript.

Flow Cytometry

Plots

Confirm that:

- [x] The axis labels state the marker and fluorochrome used (e.g. CD4-FITC).
- [x] The axis scales are clearly visible. Include numbers along axes only for bottom left plot of group (a 'group' is an analysis of identical markers).
- [x] All plots are contour plots with outliers or pseudocolor plots.
- [x] A numerical value for number of cells or percentage (with statistics) is provided.

Methodology

Sample preparation	Mouse: Lamina propria cells were isolated from the small intestine of 3d old neonatal and male adult (8-12 weeks old) mice using the Lamina Propria Dissociation Kit (Miltenyi Biotec). In brief, excised small intestines were cut open longitudinally and washed in ice-cold Hank's Balanced Salt Solution (HBSS) to remove stool. After washing, intestines were cut into <0.5cm pieces and placed into HBSS for further cleaning by brief vortexing before proceeding with manufacturer's instructions. Single cell suspensions of lamina propria cells were pelleted by centrifugation and stained with Zombie Aqua Fixable Viability Dye (Biolegend, catalog number 423102, dilution 1:200) in 1X PBS for 15min at RT. Following a flow buffer wash, Fc receptors were blocked using anti-CD16/CD32 (eBioscience, cat# 14-0161-82). Cells were then surface stained with CD4-BV786 (BD, cat# 536727, 1:400), CD45.2-FITC (eBioscience, cat# 11-0454-82, 1:200), KLRG1-BV711 (BD, cat# 564014, 1:100), NKp46(CD335)-eFluor660 (eBioscience, cat# 50-3351-82, 1:100), TCRβ-PerCPCy5.5 (eBioscience, cat# 45-5961-80, 1:500) and 1:400 of B220-PE-Cy7 (eBioscience, cat# 25-0452-82), CD11b-PE-Cy7 (eBioscience, cat# 25-0112-82), CD11c-PE-Cy7 (eBioscience, cat# 25-0114-82), CD3e-PE-Cy7 (eBioscience, cat# 25-0031-82), Gr-1-PE-Cy7 (eBioscience, cat# 25-5931-82) and TER-119-PE-Cy7 (eBioscience, cat# 25-5921-82) in ice-cold FACS buffer for 30min. Cells were then fixed using the Fixation/Permeabilization Concentrate and Diluent (eBioscience) for 30min at RT. Intracellular staining with GATA3-BV421 (Biolegend, cat# 653814, 1:25), T-bet-PE-CF594 (BD, cat# 562467, 1:25) and RORγt-PE (eBioscience, cat# 12-6988-82, 1:200) was performed in permeabilization buffer (eBioscience) for 30min at 4°C.<br><br>Human: 500μl to 3.5ml of blood samples, depending on sampling time points, were drawn from participants into sodium citrate tubes. For preterm infants, cord blood was collected at birth and peripheral blood collected between 8 and 16 hours of life (this sample is known as the day 1 sample), around weeks 1 and 2 and 36 weeks corrected gestational age from an indwelling arterial catheter or a peripheral vein. Citrated blood samples from infants or adults were centrifuged at 300xg for 15 min at room temperature (RT) onsite in hospital laboratories within 2h of sample collection, followed by removal of plasma. The remaining

blood was diluted 1:4 in culture media [RPMI 1640 (Gibco) with 1% human serum (Sigma-Aldrich, St. Louis, USA) and 1:500 Mycozap Plus-PR (Lonza, Switzerland)] and cultured in 12ml sterile polypropylene tubes (Greiner Bio-One, Austria) at 37°C, 5% CO2 overnight in the presence of 2µg/ml brefeldin A (BFA) (Sigma-Aldrich). For antibody staining, cells were washed with PBS once and incubated with eBioscience Human Fc Receptor Binding Inhibitor (Invitrogen, Carlsbad, USA) for 20min at RT. Cells were surface-stained with Fixable Viability Dye eFluor 780 (eBioscience, cat# 65-0865-14, 1:2000), CD45-BUV805 (BD, cat# 564914, 1:60), CD66b-BB515 (BD, cat# 564679, 1:40), CD3-BUV496 (BD, cat# 564809, 1:40), CD19-BV786 (BD, cat# 563325, 1:40), CD56-PECy5.5 (eBioscience, cat# 35-0567-42, 1:60), CD14-BUV395 (BD, cat# 563561, 1:30) and CD16-BV520 (BD, cat# 563830, 1:60) for 30min at RT in the dark. Thereafter, each tube was fixed and permeabilized with eBioscience FoxP3/ transcription factor staining buffer set (Invitrogen) for 30min at RT in the dark before being washed once with 1x Permeabilization Buffer (Invitrogen). IL-37-PE (eBioscience, cat# 12-7379-42, 1:50) intracellular staining was then incubated for 1h at RT. Samples were then washed once with flow buffer and stored in the dark at 4°C until acquisition.

Instrument

Mouse: BD LSR Fortessa X-20 flow cytometer (BD Bioscience)
Human: BD LSR II (BD Biosciences)

Software

FlowJo V10 software (TreeStar)

Cell population abundance

No cell sorting performed.

Gating strategy

Mouse: Gating strategy was based on FMO and single color controls. Subtyping into specific ILC groups are detailed in the Methods section of the manuscript.
Human: Gating strategy was based on FMO and single color controls.

☒ Tick this box to confirm that a figure exemplifying the gating strategy is provided in the Supplementary Information.
